# Cephalic and Limb Anatomy of a New Isoxyid from the Burgess Shale and the Role of “Stem Bivalved Arthropods” in the Disparity of the Frontalmost Appendage

**DOI:** 10.1371/journal.pone.0124979

**Published:** 2015-06-03

**Authors:** Cédric Aria, Jean-Bernard Caron

**Affiliations:** 1 University of Toronto, Department of Ecology and Evolutionary Biology, 25 Willcocks Street, Toronto, Ontario, M5S 3B2, Canada; 2 Royal Ontario Museum, Department of Natural History-Palaeobiology, 100 Queen’s Park, Toronto, Ontario, M5S 2C6, Canada; 3 University of Toronto, Department of Earth Sciences, 22 Russell Street, Toronto, Ontario, M5S 3B1, Canada; University of Oxford, UNITED KINGDOM

## Abstract

We herein describe *Surusicaris elegans* gen. et sp. nov. (in Isoxyidae, amended), a middle (Series 3, Stage 5) Cambrian bivalved arthropod from the new Burgess Shale deposit of Marble Canyon (Kootenay National Park, British Columbia). *Surusicaris* exhibits 12 simple, partly undivided biramous trunk limbs with long tripartite caeca, which may illustrate a plesiomorphic “fused” condition of exopod and endopod. We construe also that the head is made of five somites (= four segments), including two eyes, one pair of anomalocaridid-like frontalmost appendages, and three pairs of poorly sclerotized uniramous limbs. This fossil may therefore be a candidate for illustrating the origin of the plesiomorphic head condition in euarthropods, and questions the significance of the “two-segmented head” in, e.g., fuxianhuiids. The frontalmost appendage in isoxyids is intriguingly disparate, bearing similarities with both dinocaridids and euarthropods. In order to evaluate the relative importance of bivalved arthropods, such as *Surusicaris*, in the hypothetical structuro-functional transition between the dinocaridid frontal appendage and the pre-oral—arguably deutocerebral—appendage of euarthropods, we chose a phenetic approach and computed morphospace occupancy for the frontalmost appendages of 36 stem and crown taxa. Results show different levels of evolutionary decoupling between frontalmost appendage disparity and body plans. Variance is greatest in dinocaridids and “stem bivalved” arthropods, but these groups do not occupy the morphospace homogeneously. Rather, the diversity of frontalmost appendages in “stem bivalved” arthropods, distinct in its absence of clear clustering, is found to link the morphologies of “short great appendages,” chelicerae and antennules. This find fits the hypothesis of an increase in disparity of the deutocerebral appendage prior to its diversification in euarthropods, and possibly corresponds to its original time of development. The analysis of this pattern, however, is sensitive to the—still unclear—extent of polyphyly of the “stem bivalved” taxa.

## Introduction

Most stem arthropods found in the early Phanerozoic fossil record, despite limited differentiation of cephalic limbs, have diagnosable frontalmost pairs of appendages, some more antenniform, some more spinose and prehensile that are sometimes broadly referred to as “great appendages” [1–4]. Part of the “Arthropod Head Problem” (sensu [5]), these appendages have been interpreted as either pre-oral (protocerebral, deutocerebral) [5] or post-oral (tritocerebral) [4, 6–9]. Controversy remains regarding the extent to which homologies exist among “great appendages,” including the “frontal appendages” of dinocaridids (= anomalocaridids *sensu* Budd and Telford [10]), the “(short) great appendages” of megacheirans (e.g. [1, 3, 4, 11]), those of some “bivalved arthropods” [12, 13] and even the “Specialized Post-antennal Appendages” (SPAs) of fuxianhuiids [14]. The homology of the dinocaridid “frontal appendages” with megacheiran “short great appendages” in particular is pivotal in the debate. Evolutionary continuity of these appendages would not only illustrate the early evolution of the chelicerae [4, 8, 11], but, also, given a deutocerebral homology [7, 15, 16], would highlight the link between the “great appendages” *sensu lato* and the evolution of antennae/antennules in antennulate clades [7, 17–20]. By their arguably basal phylogenetic position, “stem bivalved arthropods” and their range of frontalmost appendage morphologies would be expected to yield the relevant evidence clarifying this morphological/topological transition [5, 21, 22].

Homology hypotheses have a bearing on phylogenetic matrix codings, and emphasis has been placed on the implications of different interpretations of the frontalmost appendage for the problematic relationships among early arthropods [5, 7, 8, 21]. But the evolution of morphology is also that of its variability (and realized variation at higher taxonomic levels), and the question of differences in disparity between stem- and crown-group anatomies is certainly well exemplified by frontalmost appendages in arthropods. It may seem indeed a simple observation that frontalmost appendages in stem arthropods encompass a greater morphological range than the *a priori* structurally more stable—albeit dramatically diverse—antennules/antennae and chelicerae of modern taxa, but remains difficult to discuss in lack of quantification. Although it is central in the context of the “Cambrian Explosion” and the emergence of body plans (e.g. [23–26]), disparity as a whole has been explored in a far more limited fashion than phylogenetics [27, 28]. Disparity metrics and morphospace occupation (i.e. phenetics) provide a complementary eco-functional and structural perspective on the differences between taxa (e.g. [26, 29]). Assumptions of homology also have an impact on the interpretation of a morphospace, but, similarly to a phylogeny, the implications of different hypotheses can be compared in a single analysis. In the context of this study, should a protocerebral appendage be functionally “replaced” by a deutocerebral one (see below), the relative amount of structural change that such a transition represents should be reflected in the multivariate distribution of taxa.

Hereafter we introduce the morphological evidence provided by a new “bivalved arthropod” from the recently discovered Burgess Shale locality of Marble Canyon [30] to discuss the structure and topology of frontalmost appendages. We then present a synthetic empirical morphospace of this apparatus in a sample of stem and crown-group arthropods with the purpose of quantifying morphological transitions between groups in terms of structural change—and thus estimating an eco-functional signal to be compared with the phylogenetic one.

## Frontalmost Homology: Available Evidence

The term “great appendage” was coined by Raymond in 1935 to refer to the appendages of *Leanchoilia* Walcott, which Størmer [1], following a hypothesis initially formulated by Henriksen [31], and later used in homology with both the chelicerae and the frontal appendages of *Hurdia* Walcott (at the time considered to belong to *Sidneyia* Walcott). Subsequently, Bergström [2] co-opted the terminology to describe all Cambrian arthropods with developed pleurae and undivided telson that displayed a single anteriormost prominent pair of appendages (considered, as by Størmer, to be the second antenna)—a classification later formalized by Hou and Bergström [3] under the class Megacheira. Bergström presented evidence to ally the megacheirans with crustaceans, and the link between “great appendages” and chelicerae faded into the background. Additional research on “great-appendage arthropods” from the Chengjiang Biota and Burgess Shale [4, 11], however, brought new attention to Størmer’s thesis, that is, first, the existence of structural similarities between the short, chelate version of the megacheiran appendage (“short great appendage”) and chelifores/chelicerae, and, second, the possible origin of this appendage amid the ancestral diversity of anomalocaridid frontal appendages.

We also know that comparable appendages were present in some members of the “bivalved” body plan *sensu lato* (e.g. *Occacaris* Hou [12] and *Isoxys* Walcott [13, 32]), though our understanding of these “bivalved great-appendage arthropods” is nascent. *Isoxys* has been long known, but its soft parts were only recently described [13, 32–34], and *Occacaris* has been published as a rare taxon with limited emphasis on the significance of its frontalmost appendages *per se* (whereas *Forfexicaris* Hou from the same study [12] is not clearly a different morphotype).

Alternatively, other “bivalved arthropods” and relevant stem arthropods can have post-antennular differentiated appendages with little structural resemblance with either dinocaridid or megacheiran “great appendages.” Briggs [35] considered the “claws” of *Branchiocaris* to be post-oral, and Yang et al. [14] proposed that the peculiar “hooks” of fuxianhuiids were tritocerebral, but others (e.g. [5]) have discussed the possible existence of two pairs of pre-oral appendages—a hypothesis also put forward by Scholtz and Edgecombe [7, 36], though they considered the “great appendage” to be second anteriormost, instead of first and later reduced into e.g. bilobed labrums [5]. Recent direct evidence of brain structures in fossils has suggested that in *Fuxianhuia* [37], the structures later called specialized post-antennal appendages (SPAs, [14]) were innervated above the stomodeal aperture and close to the deutocerebral neuropil. As the authors contend, such a configuration does not preclude a tritocerebral affinity, as some extant crustacean taxa such as malocrostracans also feature a bipartite tritocerebrum penetrated by the stomodaeum ([37]; Edgecombe and Strausfeld, pers. comm. 2013). In addition, fossils of cf. *Alalcomenaeus* Simonetta have featured “a large neuropil” just posterior to the protocerebrum [15], that the authors topologically interpreted as the deutocerebrum. Although in this case the differentiation between deuto- and tritocerebrum remains in question—an issue considering that those neuropils can be fused in certain arthropods (e.g. [38])—the topological position of “short great appendages” is in general consistent with a deutocerebral interpretation.

Based on their phylogenetic dataset, Legg and colleagues [9, 21, 39] recently inferred the homology of the post-antennal differentiated appendages in certain bivalved arthropods with the megacheiran “great appendages,” which they considered tritocerebral, as in Cotton and Braddy [8]. They also homologized the frontalmost appendage of e.g. *Isoxys* with the “frontal appendages” of dinocaridids [22], which they considered to be protocerebral, as in Budd [5]. The motivation for assigning a post-antennal “great appendage” to “bivalved arthropods” came in part from interpretations of anomalocaridid and “bivalved arthropod” heads in Budd [5] with respect to the protocerebral antennae of onychophorans [40], and, also, in part, from the taxa considered to be “antennulate megacheirans.” Unfortunately, clear evidence of the existence of antennules anterior to chelate “great appendages” has yet to be published. Bergström and Hou [41] in fact, cast doubt on their own interpretation of *Fortiforceps* Bergström and Hou, suggesting that the “antennae” might actually be elements from another animal; it is indeed impossible to be certain from their published material. As for *Kootenichela deppi* Legg [9], our own observations of the poorly preserved material can confirm neither the head configuration of the animal (especially the presence of a pair of antennules and the identity of the frontal appendages) nor the overall megacheiran interpretation of the anatomy. We therefore consider this taxon as an arthropod *incertae sedis*. In *Occacaris*, the “antennae” are most certainly a pair of anteriormost legs, as argued also by Vannier et al. [42].

There is, regardless, little doubt of the existence of a pair of protocerebral “limbs” in stem arthropods. For instance, a number of bivalved and other taxa (CA and JBC, pers. obs.) bear a pair of lobes between the eyes, as exemplified by *Canadaspis* [43], anterior to the antennuliform appendages *per se*. These “buds” might be the only vestigial evidence of a reduction of dinocaridid frontal appendages, but such an interpretation at this stage is speculative (hereafter, we will by default term “frontalmost” the antennular appendage instead of the pair of ocular lobes). Notwithstanding, the arguably protocerebral affinity of antennules in “higher” (or “armoured”) lobopodians (i.e. large lobopodian taxa with developed frontal appendages, as in e.g. *Megadictyon*, *Jianshanpodia*, *Kerygmachela*; see e.g. [44]), with respect to the onychophoran anatomy, questions the timing and mode of topological transition from proto- to deutocerebral innervation in frontalmost appendages [45]. Very recent evidence has been presented in favour of a protocerebral origin of anomalocaridid frontal appendages based on putative neural remains [46], although the protocerebral lobes in *Lyrarapax unguispinus* Cong et al. YKLP13305 do not constitute direct evidence for the innervation of the frontal appendages, but rather seem to correspond to the anteriormost lobes of plesiomorphic “bivalved” taxa. Despite some uncertainties, this hypothesis can be ignored no longer, as a protocerebral-deutocerebral transition arguably takes place along the arthropod stem.

In the following study, we therefore adopt the view that frontalmost/anteriormost appendages are deutocerebral throughout in euarthropods, while leaving open the question of proto- to deutocerebral transition among lobopodians/dinocaridids/“stem bivalved taxa.” Accordingly, we compare the implications for morphological change under the different hypotheses, with a focus on the role of “stem bivalved taxa.”

## Materials and Methods

### Collection and observations

The holotype and only known specimen (part and counterpart) comes from the upper part of the basinal Stephen Formation and was collected within a two metre thick interval near Marble Canyon (Kootenay National Park, British Columbia) [47]. The specimen was studied using a range of photographic techniques commonly employed for this type of material, including interference imagery [48]. Elemental maps were obtained using a FEI Quanta 200 FEG environmental scanning electron microscope equipped with an energy scanning spectroscopy (EDS) X-ray detector under low vacuum conditions (70Pa, 15Kv, 400μs dwell time) at the University of Windsor’s Great Lakes Institute for Environmental Research. All necessary permits were obtained for the described study (Parks Canada collection and research permit to JBC YNP-2012-12054), which complied with all relevant regulations.

### Morphospace analysis

Limiting the morphospace to the characters describing the frontalmost appendages allows us to interpret forms for which the rest of the body is unknown. The ability to use data from such taxa—e.g. *Caryosyntrips* Daley and Budd [49], *Amplectobelua* Hou et al. [50], and *Tamisiocaris* Daley and Peel [51]—is critical in our case given the unique morphologies of their frontalmost appendages. As pointed out by, e.g., Ridley [52], morphospaces are not designed to display logically optimal clusterings of taxa, as phylograms do, but they instead emphasize relative representations that can be used to evaluate specific hypotheses. In our case, the morphospace is meant to: 1) formalize (i.e. quantify) our anatomical perception of frontalmost appendages, 2) serve as a basis for a clarification of what “great appendages” could represent in this context, depending on their resolution, 3) evaluate the degrees of discrepancy between the distribution of all possible frontalmost appendages and the clustering expectations based on general body plans and, if possible, 4) allow us to look for quantitative changes in disparity among major groups. This latter point, when associated with a phylogenetic framework in the range of fossils for which bodies are known, can give clues about the evolution of morphological constraints, a central theme in the canalization of body plans (e.g. [29, 53–56]), and a traditional focus of debate in the case of arthropods [26, 28, 57, 58]. The results presented herein are discussed in the light of previously published topologies [5, 8, 21, 58].

From the contingency table of 36 taxa and 12 characters shown in Supporting Information (S1 Dataset) a dissimilarity matrix was produced using the *daisy* function in the *cluster* package in *R* [59]. *daisy* is a modular, ready-to-use function able to handle mixed variables (numeric, nominal and ordinal) using a generalization of the Gower index [60]. Additionally, the algorithm specifically treats missing values according to the type of data applicable in each particular column. The dissimilarity matrix was then used for principal coordinates analysis (PCoA) using the *cmdscale* function of the *stats* package in *R*. The number of most influential axes was determined using the *screeplot* function of the *vegan* package on a redundancy analysis (RDA) of the dissimilarity matrix (see S1 Fig). We used a k-means algorithm (*kmeans*, see [61]), also from the *vegan* package, to detect statistical clusters using the *cascadeKM* function, which provides the best partition for a set number of tried groups under the Calinski-Harabasz criterion [62] (see S1 Fig). Beyond eight or nine possible k-means groups, the best partition approached the largest number of possible groups, so we stopped at the first optimum, which was six groups.

To measure the degrees of association between the characters and the different principal-coordinate axes—analogous to numerical loadings in PCA—we followed Foote [63] and used Cramér’s coefficient, which is a part of the family of chi-square statistics [64], from the *assocstats* function of the *R* package *vcd* [65]. Cramér's coefficient (V) varies from 0 (no association) to 1 (complete association). After Siegel and Castellan [66], Foote preferred to apply Cramér's V to multistate unordered variables, using the gamma coefficient instead in the case of binary and ordered characters [63]. Cramér's V nonetheless has been introduced and used as a polyvalent measure of intercorrelation applicable to nominal, ordered and interval scaled variables [64, 67], and we chose in this case to use it on our entire dataset. After Kotrc [68], we also extracted the p-values of chi-square tests using, as for Cramér's V, the *assocstats* function of the *R* package *vcd* [65]. Like Foote [63] and Kotrc [68], we also created the necessary contingency tables for those tests by dividing each axis into four intervals of equal length. We then combined this information for each axis in the form of two superimposed pie charts: an inner pie composed of the relative p-values of the significant characters (95% threshold), and an outer ring displaying the corresponding Cramér's V for those significant characters.

### Comparisons of disparities

A common and effective way to measure disparity is to use sums of morphological variances [29], which works well for large, uniform groups that can be grouped into bins by, for instance, time. In our case, it was of interest to preserve the variance of groups along each axis of the PCoA, as the variance of variances (i.e. spread of variances) on different axes provides information about the discrepancies in plasticity between major traits themselves (and those proved indeed to be occasionally very large). We thus extracted the variances on each PCoA axis for each group and plotted them all in violin plots (i.e. box plots made of Kernel density distributions).

### Nomenclatural acts

The electronic edition of this article conforms to the requirements of the amended International Code of Zoological Nomenclature, and hence the new names contained herein are available under that Code from the electronic edition of this article. This published work and the nomenclatural acts it contains have been registered in ZooBank, the online registration system for the ICZN. The ZooBank LSIDs (Life Science Identifiers) can be resolved, and the associated information viewed, through any standard web browser by appending the LSID to the prefix “http://zoobank.org/.” The LSID for this publication is: urn:lsid:zoobank.org:pub:B3B1A160-8D1A-4F42-AEE1-C1B1DC940C58. The electronic edition of this work was published in a journal with an ISSN, and has been archived and is available from the following digital repositories: PubMed Central and LOCKSS.

### Institutional abbreviations

MGUH: Museum Geologicum Universitatis Hauniensis, Copenhagen, Denmark; MNHN: Naturhistorisches Museum/Landessammlung für Naturkunde, Mainz, Germany; ROM: Royal Ontario Museum, Toronto, ON, Canada; USNM: National Museum of Natural History, Washington, DC, USA; YKLP: Yunnan Key Laboratory for Paleontology, Kunming, China.

## Systematic Palaeontology

**Superphylum Panarthropoda Nielsen, 1995**

**Phylum Arthropoda Siebold, 1848 [69]**

**Order Isoxyda Simonetta & Delle Cave, 1975**

**Family Isoxyidae Vogdes, 1893**

= Isoxysidae Brooks and Caster, 1956

### Diagnosis (amended from Brooks and Caster 1956 to include soft parts)

Bivalved panarthropods with poorly segmented bodies, eyes rounded and large relative to body length (ca. 15% of carapace length excluding cardinal processes) and a single pair of prominent, sub-straight to upward-directed frontalmost appendages. Carapace semi-circular with attachment to body proper located anterodorsally. Antero- and posteromedial margins of carapace sometimes prolonged into rostral processes that can be up to 140% of the length of the carapace itself [70]. Posterior tailpiece protruding slightly beyond carapace, may take the form of a lobe fan [22]. Anterior portion of the animal likewise slightly jutting out of carapacal margin. Frontalmost pair of appendages stout, differentiated into a predatory antennule, composed of five or more segments, with spines of variable length and arrangement on the inner margin and sometimes on the terminal outer margin. Trunk limbs biramous.

### Genera included

*Isoxys* Walcott, *Surusicaris* Aria and Caron gen. nov.

### Type genus

*Isoxys* Walcott.

**Genus *Surusicaris*** Aria and Caron **gen. nov.** urn:lsid:zoobank.org:act:2D65C133-BA27-47D6-8D3D-DC9F962FC243

### Type species

*Surusicaris elegans*, by monotypy.

### Etymology

After *Surus* (“The Syrian”), purported last war elephant of Hannibal, described by Pliny the Elder as bearing a single tusk and a shield, referring to the lateral habitus of the animal, including carapace and spinose trunk-like frontalmost appendages; and καρίς, the Greek for “shrimp” or more generally “kind of crustacean” in the Latin “caris.”

### Occurrence

Upper basinal Stephen Formation, Marble Canyon (Kootenay National Park, British Columbia) (30).

### Diagnosis

Bivalved arthropod with the following characters: carapace with smooth margins and no cardinal processes, body 16-segmented, divided into anterior (four-segmented) and posterior tagmata (12-segmented); head protruding anteriorly, with a pair of large eyes; frontalmost pair of appendages dorsally oriented and composed of five main segments bearing spinose outgrowths on their inner margin, with distalmost article ending in a set of three main elongate spines inserted on the outer margin and distalmost segment possibly subdivided into three shorter segments; three post-oral pairs of short and thick uniramous limbs, weakly sclerotized, ending in a small bifid claw; trunk limbs biramous, with thick, poorly segmented endobasipod broadly attaching to elongate filamentous exopod branch.

### Remarks

The presence of a carapace with no cardinal processes justifies erection of a new genus within the family Isoxyidae; all *Isoxys* species have cardinal processes. Number of segments, morphology of anterior and posterior limbs, as well as the morphology of the frontalmost appendage cannot at present be compared to all *Isoxys* morphospecies, in which these characteristics are generally unclear. No published *Isoxys* morphotype exhibits an upward-directed frontalmost appendage with an ornamentation similar to that of *Surusicaris*, although such animals may be already known (see discussion below).

**Fig 1:**
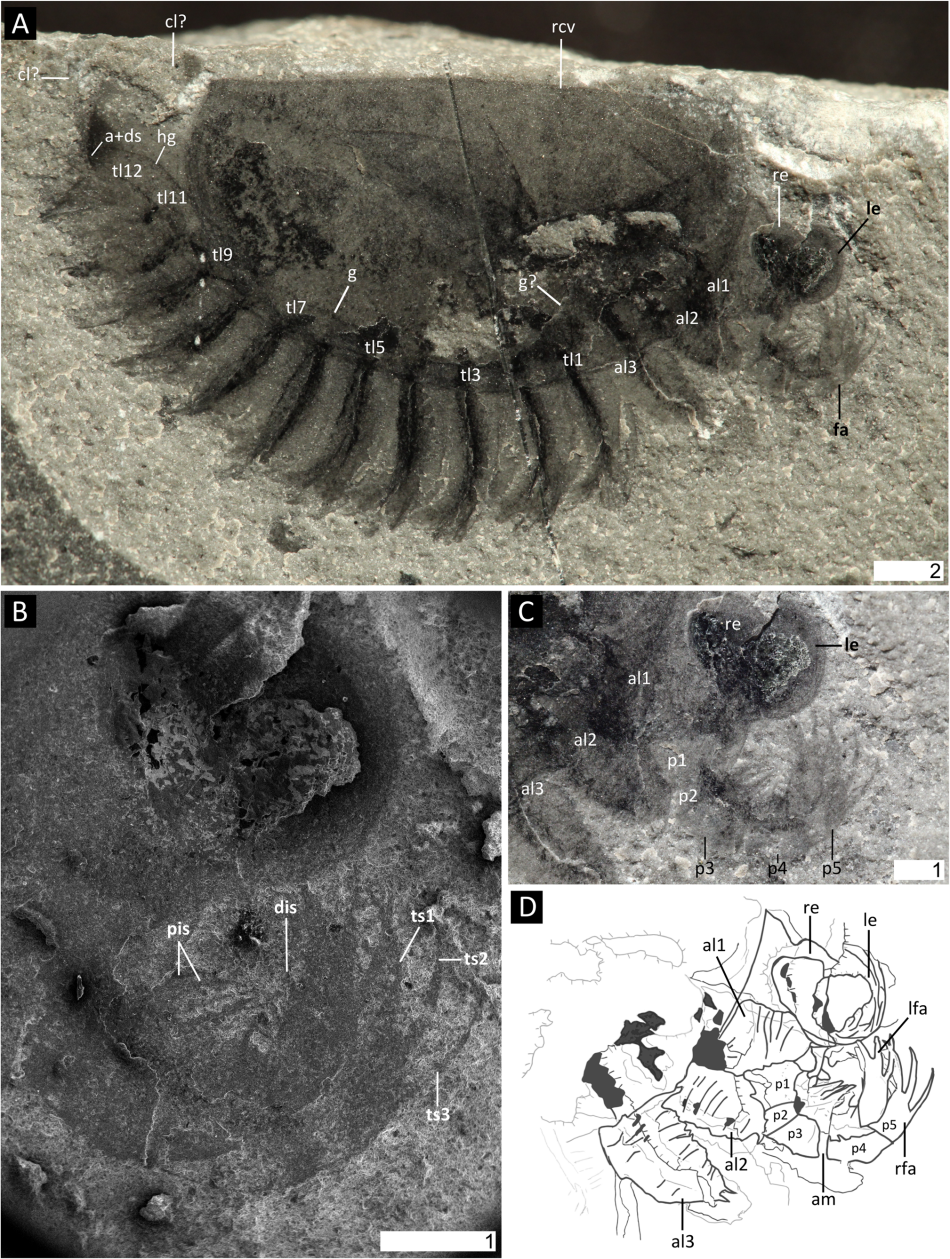
*Surusicaris elegans* gen. et sp. nov., holotype specimen ROM 62976. **A.** Complete view of the part. **B.** Secondary electron image of anterior area showing details of eyes and frontalmost appendages. **C.** Close-up of anterior section showing the three pairs of anterior uniramous legs. **D.** Camera lucida drawing of the “head.” All images were taken under cross-polarized light except in B. **Abbr.** a+ds: anus+dark stain; al*x*: anterior limb (1–3); cl?: caudal lobe?; dis: distal inner spine; fa: frontalmost appendage; g(?): gut(?); hg: hindgut; lfa: left frontalmost appendage; le: left eye; p*x*: podomere (1–5); pis: proximal inner spines; rfa: right frontalmost appendage; rcv: right carapacal valve; re: right eye; tl*x*: trunk limb (1–12); ts*x*: terminal spine (1–3). Scale numbers in mm.

**Fig 2:**
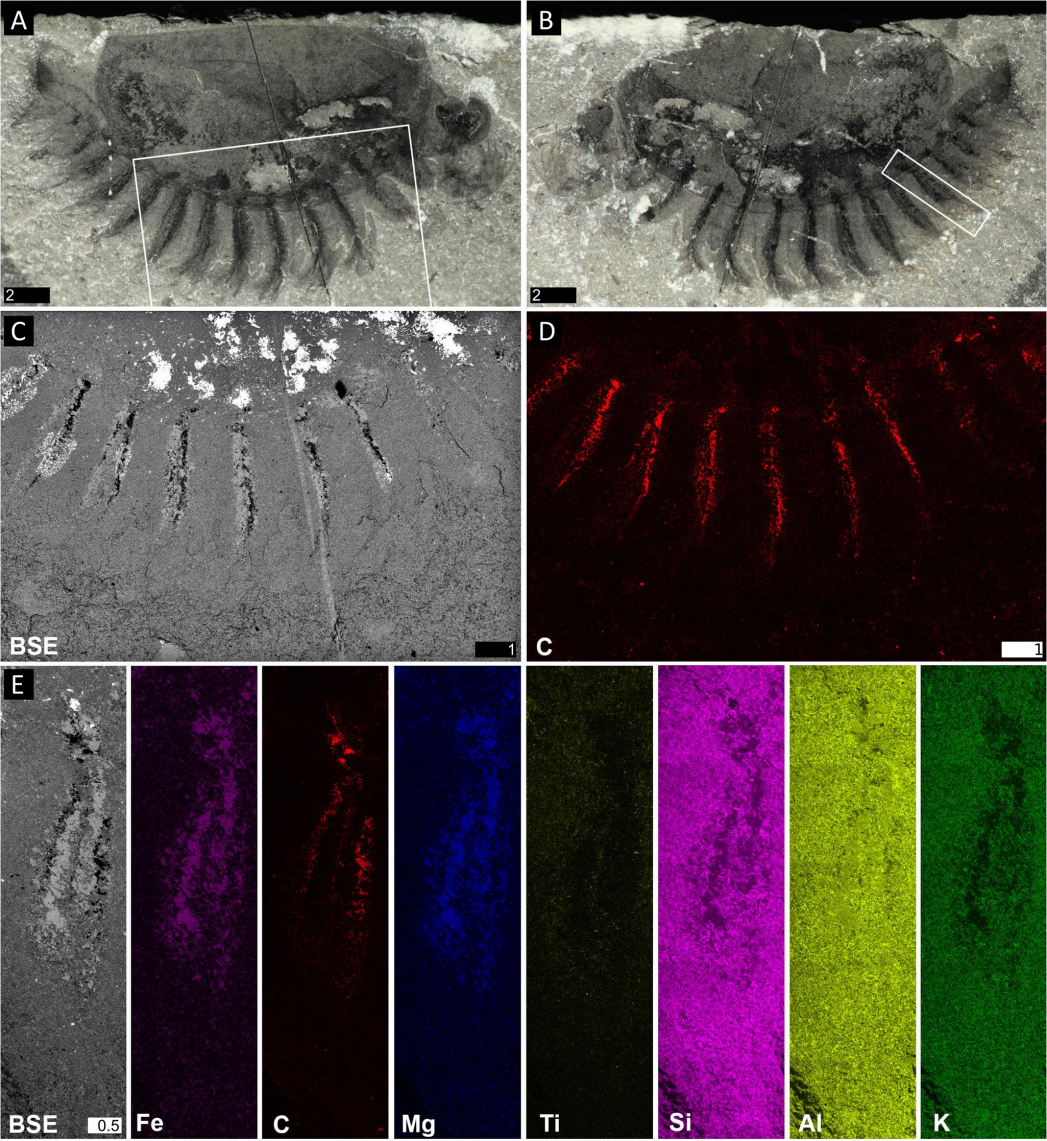
Elemental maps of *Surusicaris elegans* gen. et. sp. nov. holotype specimen ROM 62976. **A.** Part. **B.** Counterpart. **C.** Backscatter image of the insert in A, showing the conspicuous preservation of the exopods. **D.** Carbon mapping of the same area. **E.** Backscatter image and elemental maps of various elements on a single posterior exopod (insert in B). Note the exact overlap of Fe and Mg in the area between the caeca. A, B, images taken under cross-polarized light. Scale numbers in mm.

**Fig 3:**
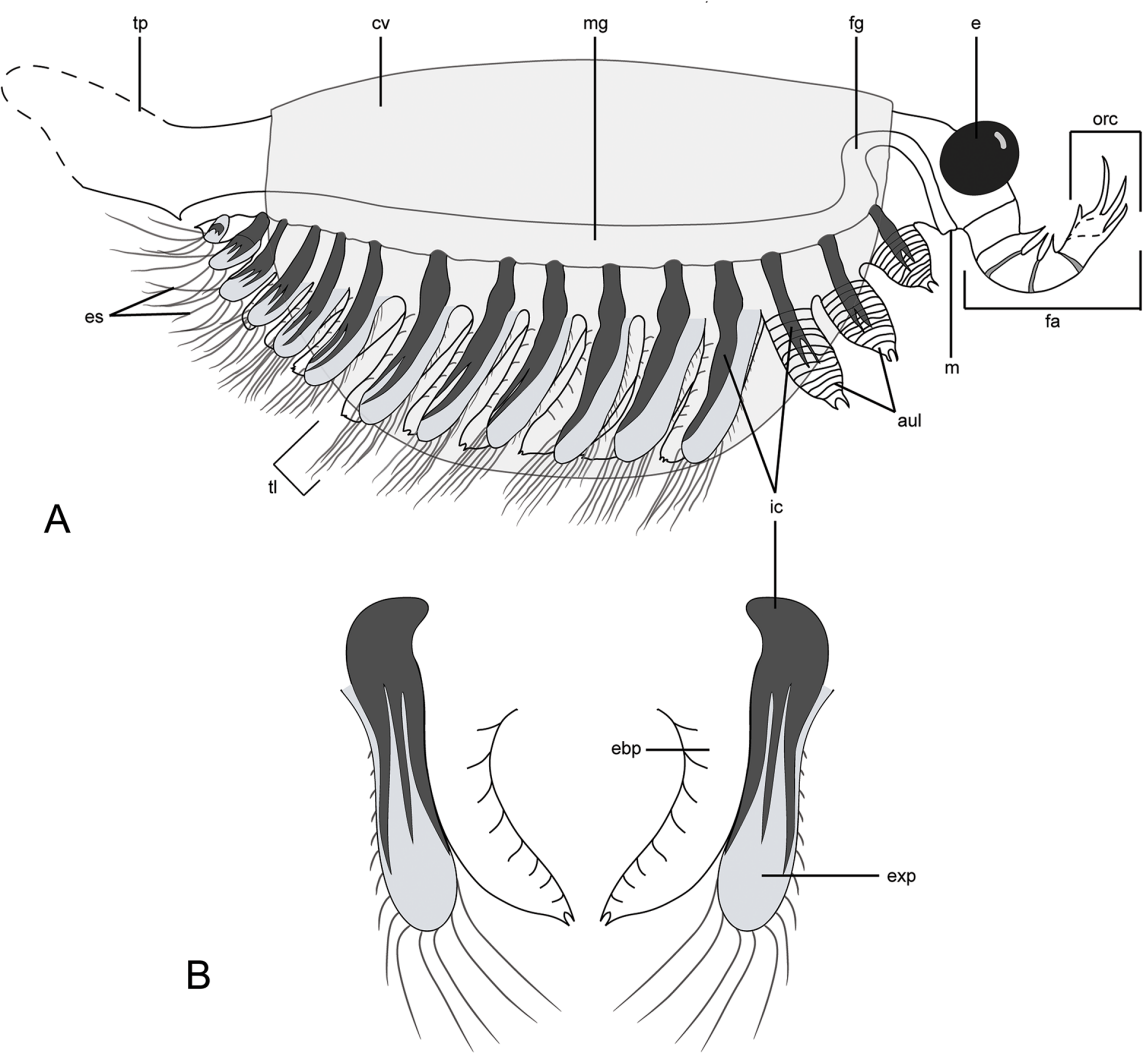
Diagrammatic reconstruction of *Surusicaris elegans* gen. et sp. nov in profile view. For clarity, exopods are figured in light grey and caeca in black. **A.** Habitus. Only the right appendages are drawn and the distalmost segment of the frontalmost appendage is here hypothetically subdivided into three additional segments, based on the anomalocaridid morphology. Exopods are appressed onto the endopod posteriorward to show the tripartite branching of the caeca. The tailpiece is conjectural. **B.** Antero-posterior view of trunk limbs, with exopod opened up. Abbr. aul: anterior uniramous limbs; cv: carapacal valve; e: eye; ebp: endobasipod; es: exopodial setae; exp: exopod; fa: frontalmost appendage; fg: foregut; ic: invasive caeca; m: mouth; mg: midgut; orc: outer raptorial complex; tl: trunk limb; tp: tailpiece.

**Fig 4:**
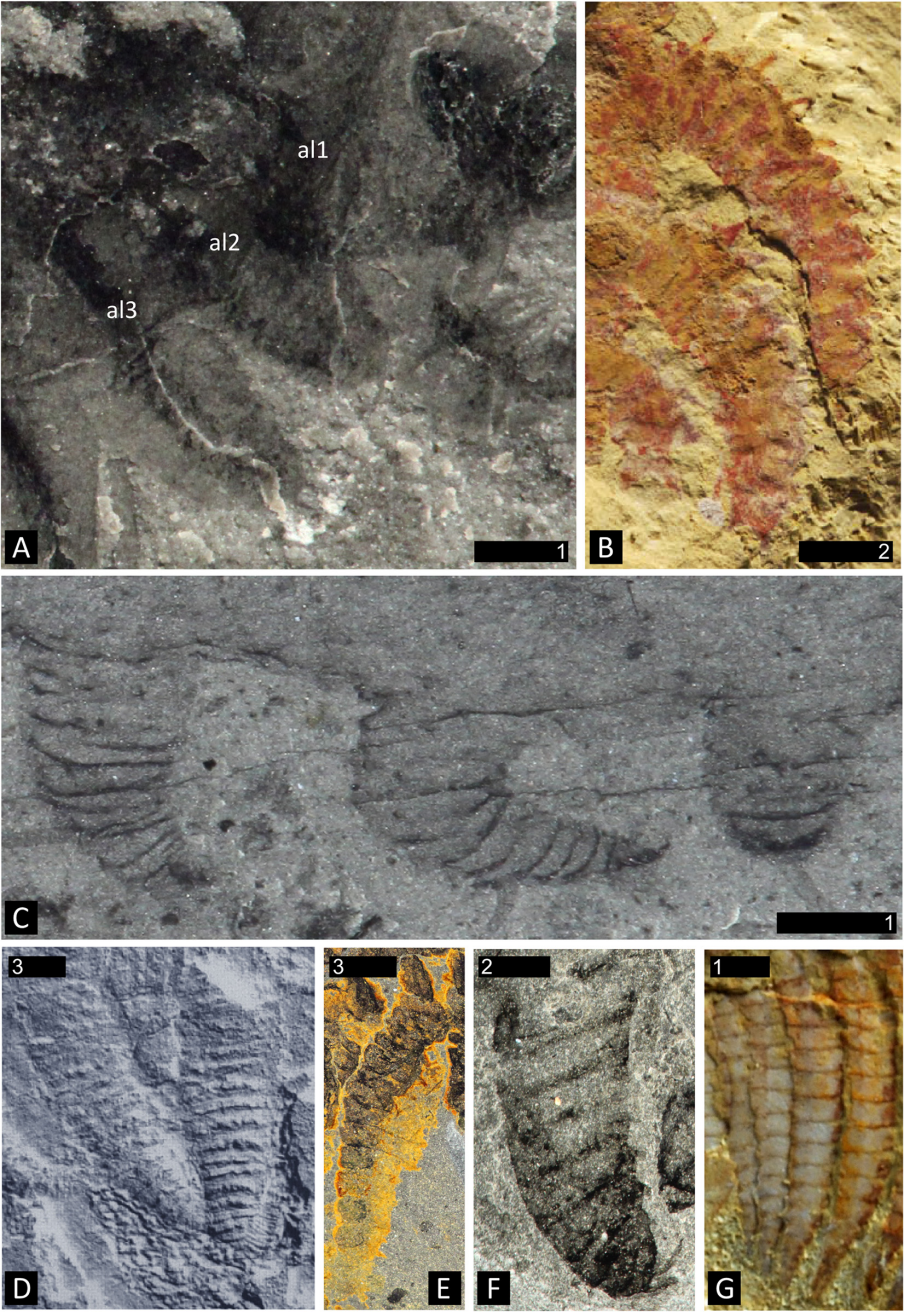
Degrees of sclerotization in lobopodous and stenopodous limbs of fossil lobopodians and arthropods. **A.** *Surusicaris elegans* gen. et sp. nov., holotype (ROM 62976), showing the three right anterior uniramous limbs with faint traces of cuticular boundaries. **B.** Limbs of *Diania cactiformis* Liu et al., counterpart of YKLP 11319 (from [79]). Note the thickness and regularity of the subdivisions. **C.** *Aysheaia pedunculata* Walcott, part of holotype (USNM 57655), posterior limbs preserved at various angles. The aspect of the annulations varies from discordant through faint to regular. **D.** *Hadranax augustus* Budd and Peel [126], trunk lobopods of the mid-section of holotype (MGUH 24.527). **E.** Posterior endopod of the bivalved arthropod *Odaraia alata* Walcott. Poor sclerotization can lead to a deformed preservation of segments’ shape, but the rectangular aspect of some segments and the general elongated habitus usually remain characteristic of arthrodization. **F.** Anterior limb of *Canadaspis perfecta* preserved twisted and dislocated. In certain cases, taphonomy can reshape an arthrodized limb to a much more compact structure, although the undulation or folding of segments’ boundaries are not necessarily associated with such deformations. **G.** The “poorly sclerotized” anterior endopods of the fuxianhuiid *Chengjiangocaris kunmingensis* Yang et al. [14] (YKLP 12024). Note their relatively advanced arthrodization in comparison to *Surusicaris*. Abbr. al*x*: anterior limb (1–3). Scale numbers in mm.

**Fig 5:**
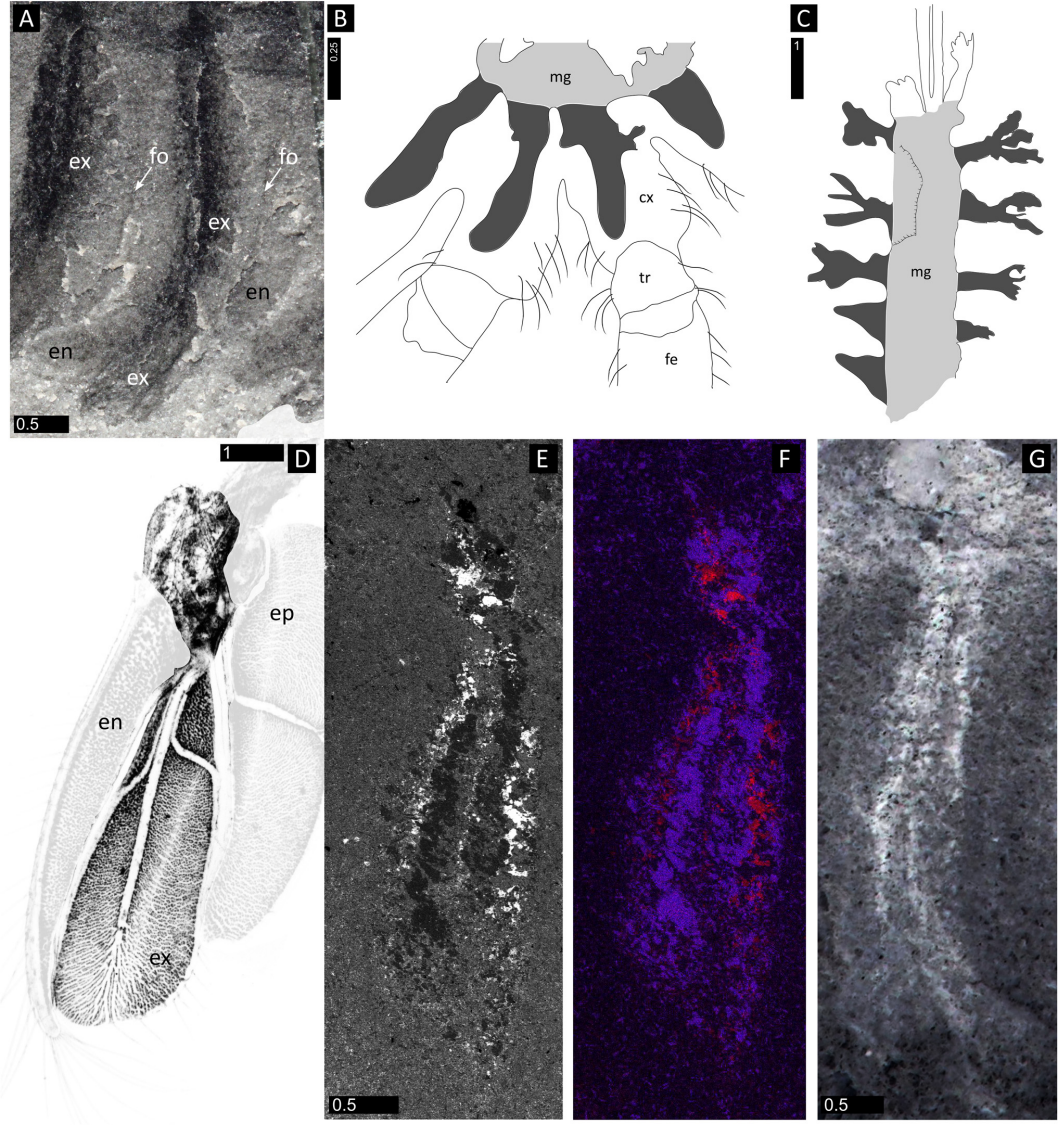
Comparative nature of the biramous trunk limbs in *Surusicaris elegans* gen. et sp. nov. A, E-G. *Surusicaris elegans*, holotype (ROM 62976). **A**. Close-up of trunk limb 3. **B.** Blind caeca (dark grey) projecting from the midgut (light grey) into the coxae in a wolf spider (redrawn from Foelix [90]). **C.** Possible equivalents in the trilobitomorph *Misszhouia longicaudata* Zhang and Hou (redrawn from Vannier and Chen [85]). **D.** 6^th^ thoracopod (left side) of the leptostracan (Crustacea: Malacostraca) *Dahlella caldariensis* Hessler (from Shu et al. [18], original picture provided by Jean Vannier). The central, exposed, part of the figure shows the exopod (below) as an anastomosed tissue spread out in-between a trident of hemolymph channels, and the mostly muscular attachment of the limb to the body (above). The epipodite and its musculature have been attenuated, as they seem to be absent on the limbs of *Surusicaris*; we have also faded the endopod, as its trace is too shallow to be revealed under SEM observation (see E and F). **E.** Backscatter image of the 8^th^ trunk limbs of the counterpart of the holotype (see also Fig 2). **F.** Superposed elemental maps of E: carbon in red, iron in magenta and magnesium in blue (Fe and Mg match exactly). Note the analogy with D. **G**. *Surusicaris elegans*, 5^th^ trunk limb (Image flipped horizontally, with inverted grayscale). B, G, images taken under cross-polarized light. **Abbr.** cx: coxa; en: endopod; ep: epipod(ite); ex: exopod; fe: femur; fo: fold; mg: midgut; tr: trochanter. Scale numbers in mm.

***Surusicaris elegans*** Aria and Caron **sp. nov.** urn:lsid:zoobank.org:act:3D87F1AF-8DA6-4CC2-8EFB-D57FDD2928D8 (Figs 1, 2, 3, 4A, 5A and 5E–5G)

### Synonymy

2014 “new isoxyid arthropod (new arthropod B),” Caron, Gaines, Aria, Mángano and Streng, p. 4, Fig 3m.

### Etymology

After the Latin, referring to the delicate spread of the trunk limbs.

### Type

The description is based on the holotype (ROM 62976, part and counterpart, Figs 1, 2, 3, 4A, 5A and 5E–5G) and only known specimen of this species, which is housed in the collections of Invertebrate Palaeontology at the Royal Ontario Museum.

### Preservation

As in other Burgess Shale-type deposits, Marble Canyon specimens are preserved primarily as carbonaceous compressions replicated to a lesser or greater extent by aluminosilicate and other minerals ([71–73]; Fig 2 herein). The only known specimen of *Surusicaris* gen. et sp. nov. (ROM 62976) is preserved laterally, and the low position of the trunk appendages (and hence of the trunk) suggests a displacement of the body relative to the dorsal hinge of the carapace; see Orr et al. [74] for relevant taphonomical scenarios.

### Diagnosis

As for the genus.

### Description

#### Habitus

Body (length [of carapace]: 14.9 mm; height [of carapace]: 8.9 mm) mostly covered by a carapace folded along the dorsal margin so as to form two smooth and lightly sclerotized semicircular valves; antero- and posterodorsal angles unadorned, i.e. smooth with cardinal processes absent (Figs 1A, 2A, 2B and 3A). Head with a large pair of eyes (ca. 15% and 25% of carapace length and height, respectively) and frontalmost appendages protruding anteriorly (Figs 1, 2A, 2B, 3A and 4A). Limb-bearing trunk extending beyond the posterior margin of the carapace, ending in a post-anal tailpiece (Figs 1A, 2A, 2B and 3A); total length and termination unclear. Trunk appendages jutting out beneath the ventral margin of the carapace and visible in lateral view (Figs 1A, 2A, 2B and 3A).

#### Frontalmost portion

Short, bearing a pair of round, prominent eyes and differentiated, segmented appendages (Fig 1A). Location of mouth opening not preserved but possibly at the base of the great appendages before the first pair of uniramous limbs. Eyes attached antero-laterally, pedunculation unclear (Figs 1, 3 and 4A).

#### Frontalmost appendage

Inserted immediately beneath the eyes (Figs 1, 2A, 2B, 3A and 4A) and fully arthrodized. Small relative to eye size, directed upward so that the apex reaches eye level. Five segments visible on the holotype (Figs 1C, 1D and 3A). Base—the large proximalmost portion is considered a segmental base, attachment to body concealed—trapezoidal in shape (p1), wider at its posterior margin; fourth distalward segment (p4) slightly longer than the two preceding ones (p2 and p3); distalmost segment (p5) slightly curved inward and subequal in length to second distalmost (p4). Distalmost (p5) segment adorned with three long spinose projections (as long as, to slightly longer than, the bearing segment) aligned on the outer apical margin and curving inward (compare with, e.g. *Anomalocaris* [75]); presence of a least one more spine on the inner apical margin, making a total of three main plus one secondary apical spinose projections. Inner margin of the three post-basal segments (p2-p4) adorned with spinose projections, albeit shorter and stouter than those of the previous segments, and with elongate triangular outline. The exact configuration of the attachment of the projections on the appendage is not certain, and therefore the presence of only one spine at the distal portion of each segment is putative, and adornment on inner margin of distalmost segment (p5) uncertain. Distalmost segment (p5) potentially subsegmented based on the number of distal claws—see discussion—but no subsegmental boundary visible on the holotype.

#### Anterior limb-bearing section

Defined by three uniramous pairs of short and thick unarthrodized limbs, the bases of which are concealed under the carapace (Figs 1A, 1C, 1D, 2A, 2B, 3A and 4A); integument weakly sclerotized so that “segment” shape varies; presence of at least 12 “segments” or annulations, significantly wider than long; distal tip a small bifid claw (seen preserved only in al2 and al3). Slightly increasing in size posteriorward.

#### Trunk limbs

12 trunk limb pairs: the first six pairs are subequal in size and the following six pairs taper posteriorward. Limb biramous, phyllopod-like—though extremely simple (Figs 1A, 2, 3B, 4A, 5A and 5E–5G); endobasipod relatively thick, preserving similarly to the anterior uniramous appendages, with extremely faint traces of external segmentation or annulation, broadly attached to the exopod and possibly ending in a claw; setation unclear. Exopod elongate, sub-lobate, possibly flattened, subequal to or slightly longer than endobasipod; outer margin setose, with longer setae seemingly distal. The whole limb is ca. 1/3rd longer than anteriormost legs.

#### Internal organs, Intestinal tract

Mostly preserved as a faint outline running from the anus along the postero-ventral side of the trunk; based on the position of the internal limb features to which it appears to be related (see below), the intestine possibly loops anteriorly, hence the putative mouth being just posterior to the frontalmost appendages (Figs 1A, 1C, 1D, 3A and 4A).

#### Traces of ramified tissue within the limbs

Thick carbonaceous traces adjoin the gut and run longitudinally within each trunk limb (Figs 2E and 5E–5G). The disposition of some posterior limb pairs show that the tissue divides into three main longitudinal branches most likely spread within the exopod (Fig 3).

#### Posterior end

Posterior to the anus is a tailpiece possibly constituted by two lobes or a fan (Figs 1A, 2A, 2B and 3A). Details are unclear.

## Morphospace Results

### Distribution of taxa

**Fig 6:**
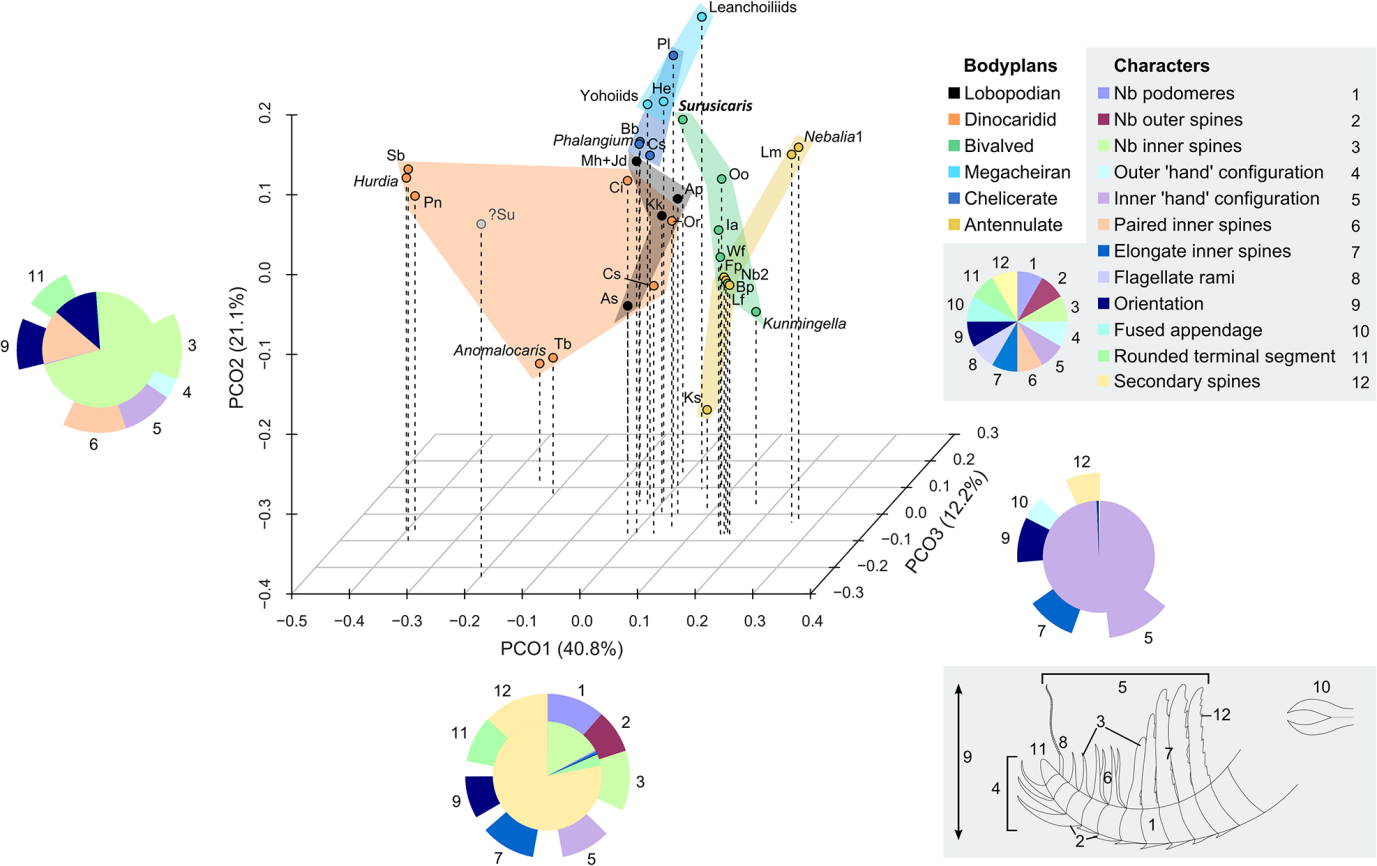
Morphospace defined by the first three axes of a PCO analysis of the frontalmost appendage. Analysis based on a matrix of 36 taxa and 12 characters (see Methods and S1 Dataset). Percentage of total variance explained by each axis displayed next to axis names. The pie diagrams describe the relative influence of the characters on each axis (see distribution pie, bottom left to the character list). The outer ring displays the proportional value of the Cramér index represented by all characters having a significant impact on the axis (p-value ≤ 0.05). The inner pie displays the proportional p-value for those significant characters, quantifying their impact on the ordination of the axes. The identity of characters used is shown on a hypothetical synthetic appendage in the bottom right corner. Abbr. *Anomalocaris*: *Anomalocaris canadensis*; As: *Amplectobelua stephenensis*; Ap: *Aysheaia pedunculata*; Bb: *Branchia brevis*; Bp: *Branchiocaris pretiosa*; Cf: *Cupiennius foliatus*; Ci: *Cassubia infercambriensis*; Cs: *Caryosyntrips serratus*; Fp: *Fuxianhuia protensa*; He: *Haikoucaris ercaensis*; *Hurdia*: *Hurdia victoria*; Ia: *Isoxys acutangulus*; Jd: *Jianshanpodia decora*; Kk: *Kerygmachela kierkegaardi*; *Kunmingella*: *Kunmingella maotianshanensis*; Ks: *Kiisortoqia soperi*; Leanchoiliids = *Actaeus armatus*, *Alalcomenaeus cambricus*, *Leanchoilia superlata*; Li: *Lithobius forficatus*, Lm: *Lightiella monniotae*; Mh: *Megadictyon haikouensis*; *Nebalia*: *Nebalia bipes* (Nb1 refers to coding of three rami, Nb2 to a single one); Oo: *Occacaris oviformis*; Or: *Opabinia regalis*; Pl: *Pycnogonum litorale* (extant); Pn: *Peytoia nathorsti*; *Phalangium*: *Phalangium opilio* (extant); Sb: *Schinderhannes bartelsi*; *Surusicaris*: *Surusicaris elegans*; ?Su: *Sanctacaris uncata*; Tb: *Tamisiocaris borealis*; Wf: *Waptia fieldensis*; Yohoiids = *Fortiforceps foliosa*, *Yohoia tenuis*.

The empirical morphospace we obtained (Fig 6) is unevenly occupied. The *Hurdia*-like and the *Anomalocaris*-like frontal appendages stand aside from the central cluster, and the lower section of axis 2 is largely vacant. Both gap and distance between *Hurdia* and *Anomalocaris* structures emphasize their strong functional divergence, as was retrieved phylogenetically by e.g. Vinther et al. [76] and Cong et al. [46].

Despite their scattered distribution in our empirical morphospace plot (Fig 6), body plans (or taxonomic groups) associated with the plotted appendage morphologies exhibit limited overlap. Notwithstanding some intersections (emphasized by the two-dimensional colour-based marking of body plans in Fig 6), lobopodians (LOB), dinocaridids (DIN), stem bivalved arthropods (BIV), megacheirans (MEG), chelicerates (CHE) and artiopods/mandibulates (“antennulates” sensu stricto, AMA) occupy their own more or less extended volumes of morphospace (Fig 6). The combinations of characters defining the axes of the tridimensional morphospace therefore bear a systematic and partly phylogenetic signal, superimposed on the functional disparity.

**Fig 7:**
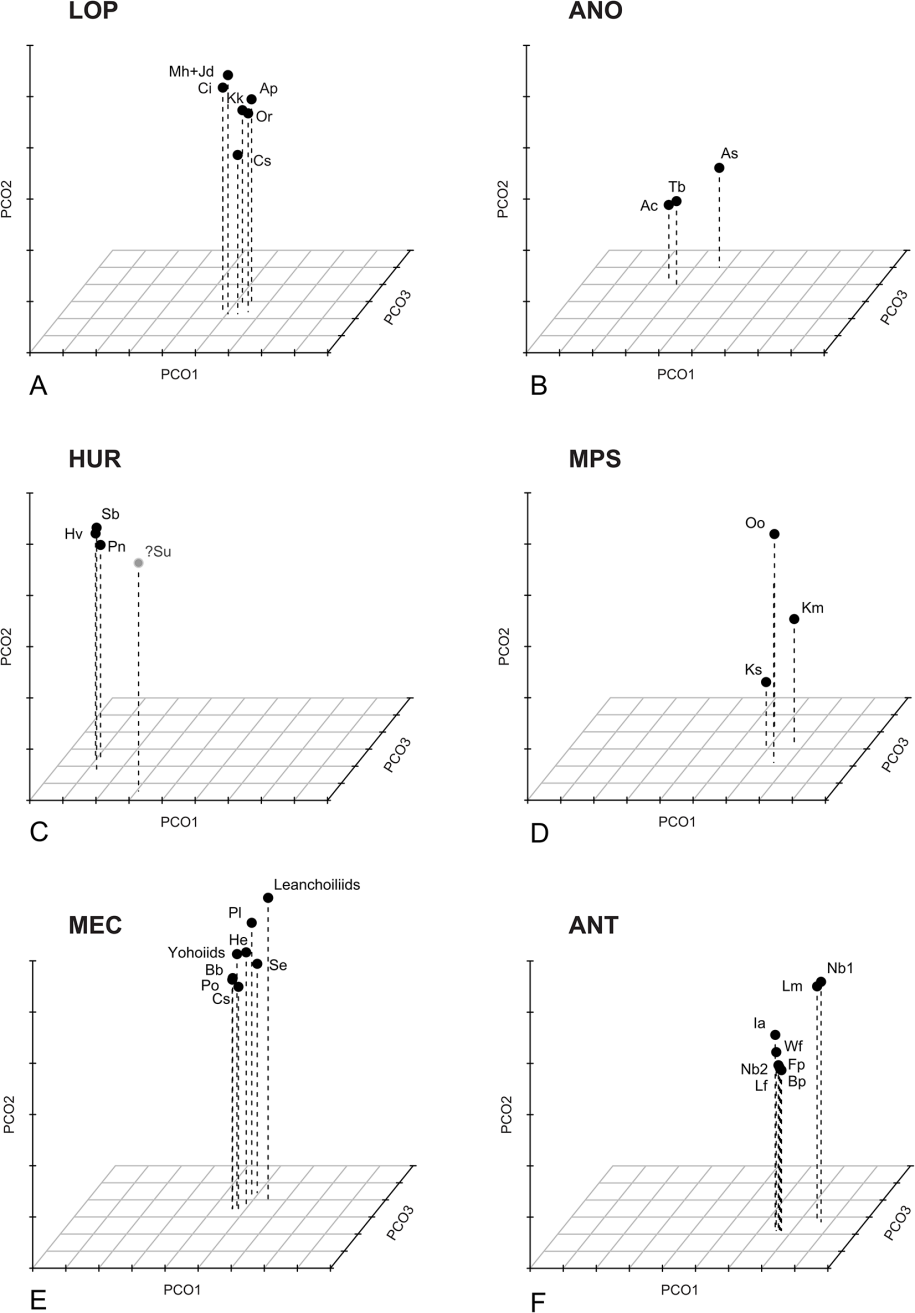
Decomposition of the tridimensional morphospace of the frontalmost appendages. Optimal clusterings found by the k-means analysis of the morphospace constrained by the Calinski criterium. **A.** LOP cluster (lobopodians, opabiniids and *Cassubia*). B. ANO, “*Anomalocaris*-type” cluster. **C.** HUR, “*Hurdia*-type” cluster (and tentatively, *Sanctacaris*). **D.** MPS, intermediate morphologies of *Kiisortoqia*, *Kunmingella* and *Occacaris*. **E**. MEC, megacheirans and chelicerates. **F**. ANT, antennulate morphologies.

The k-means clustering method recognizes six significant groups (Fig 7): the lobopodian/opabiniid cluster (LOP), the *Hurdia* cluster (HUR, to which is also attached the isolated *Sanctacaris*), the *Anomalocaris* cluster (ANO), the antennulate cluster (ANT), the mixed paired-spines cluster (MPS) and the megacheiran/chelicerate cluster (MEC). These groups transcend body plan-based boundaries between appendages and better reflect the relative discrepancies in disparity, but they remain constrained by possible phylogenetic relationships. Thus, consistently with various cladistic hypotheses [5, 8, 21, 77], opabinid appendages are allied with lobopodian, and megacheiran with chelicerate. The separate grouping of *Anomalocaris*- and *Hurdia*-like appendages could also reflect a monophyletic signal [77], also suggesting that each of these clades have evolved disparities in their frontal appendages that could be equivalent to those of large euarthropod clades.

**Fig 8:**
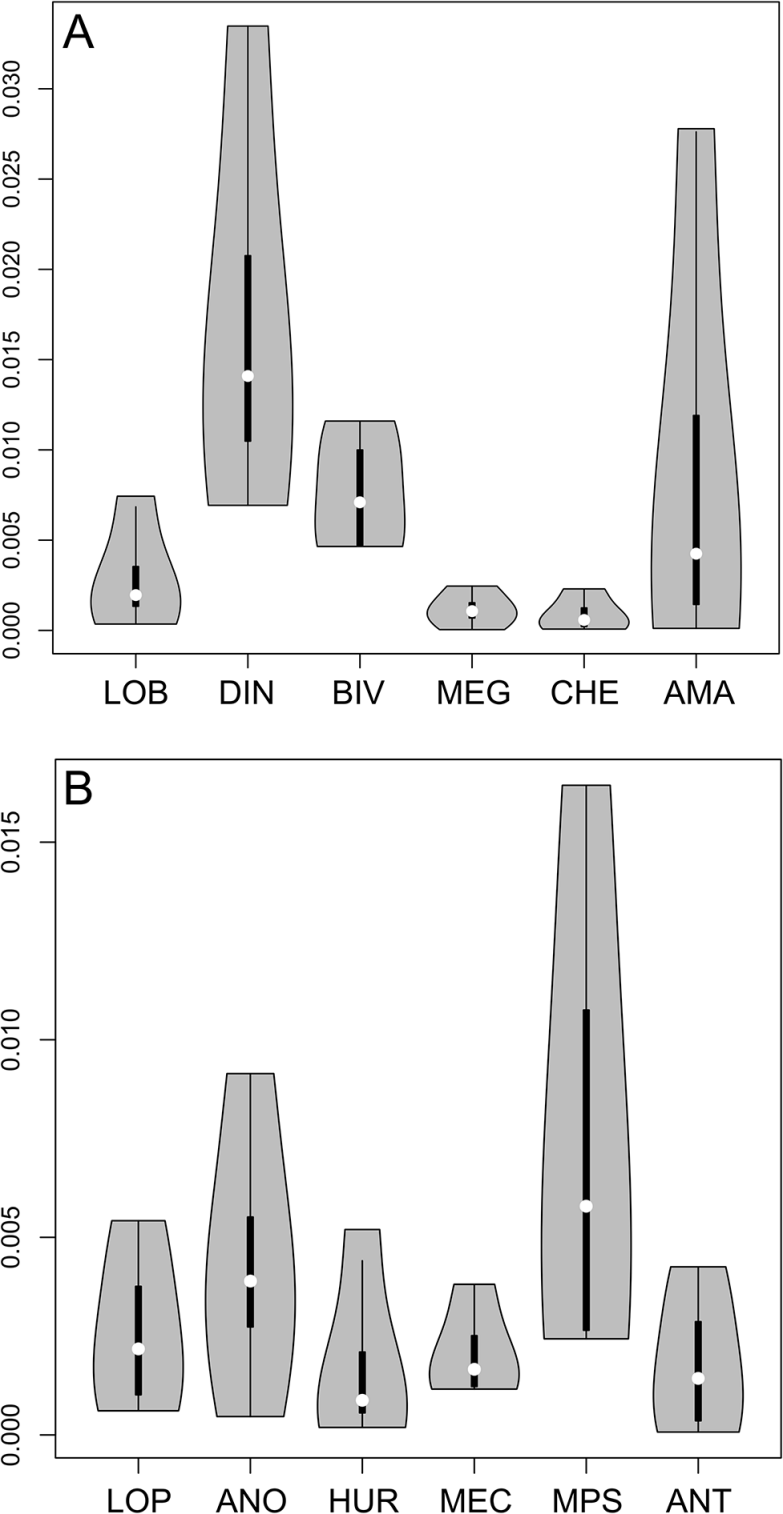
Disparity, measured as the variance of PCoA axes variances, of *a priori* and tested morphospace groups. **A.** Disparity between body plan-based clusters. **B.** Disparity between k-means-based clusters. See text and Fig 7 for description of acronyms.

Variances of variances (spread of variances) on all four significant axes in both grouping approaches (empirical body-plan based and k-means disparity based) are compared in Fig 8 (see also Methods). From a body plan perspective (Fig 8A), dinocaridid frontal appendages have the largest median disparity, followed by stem bivalved arthropods. Other groups have very low, sub-equal values of median disparity, with the exception of the artiopod/mandibulate (antennulate s.s.) taxa, which are here intermediate. The median disparity of the later is inflated by the morphologies of Cephalocarida and *Nebalia* (in its alternate coding considering several rami composing its antennule). However, the values for both bivalved and antennulate s.s. remain conservative, as *Nebalia* and *Kunmingella* could otherwise be considered to belong to both groups. The contribution of the different axes to the total disparity of antennulate s.s. (AMA) varies dramatically (Table 1), which means that certain combinations of characters are highly plastic, while others are more constrained. This situation is similar for dinocaridids, as the variance on axis 1 is much greater than on other axes, and the most significant characters on this axis (Fig 6 and Table 1, see below) are responsible for most of the morphological plasticity in the dinocaridid frontal appendage. In contrast, chelicerae and “short great appendages” are both defined by combinations of characters that vary little on all axes.

**Table 1:** Top: Values of Cramér's V calculated on the first four PCoA axes; Bottom: P-values of chi-squared tests calculated on the first four PCoA axes.

	Cramér's V				P-values of χ^2^			
	Axis 1	Axis 2	Axis 3	Axis 4	Axis 1	Axis 2	Axis 3	Axis 4
Nb. podomeres	**0.818526**	0.731247	0.746308	0.659446	**0.005973**	0.096213	0.064859	0.391902
Nb. outer spines	**0.626308**	0.409058	0.421054	**0.517448**	**0.000983**	0.450956	0.382829	**0.049399**
Nb. inner spines	**0.851003**	**0.836332**	**0.699677**	**0.672994**	**3.56E-06**	**8.47E-06**	**0.006126**	**0.016032**
"Outer hand"	0.397562	0.262643	0.205971	**0.499971**	0.077318	0.548095	0.80197	**0.006237**
"Inner hand"	**0.700309**	**0.643216**	**0.738228**	**0.601938**	**0.000139**	**0.001906**	**1.90E-05**	**0.009466**
Inner paired	0.422953	**0.799802**	0.434323	0.449171	0.092059	**3.98E-05**	0.078869	0.063967
Elongate	**0.775605**	0.353553	**0.560761**	0.216126	**7.69E-05**	0.21229	**0.010114**	0.641037
Flagellate	0.221108	**0.54917**	**0.5**	0.288371	0.623678	**0.012524**	**0.029291**	0.3926
Orientation	**0.595663**	**0.640836**	**0.514913**	0.409967	**0.00027**	**4.75E-05**	**0.004015**	0.059748
Fused	0.21693	0.214985	0.271163	**0.542326**	0.638242	0.644997	0.449299	**0.014174**
Rounded tip	**0.664455**	**0.576387**	0.354997	0.370135	**1.79E-05**	**0.00054**	0.169479	0.130497
Secondary spines	**0.930949**	0.421637	0.394565	0.220048	**7.71E-07**	0.093691	0.132518	0.627382

Note that high Cramér's V are not necessarily found to be significant (p-value ≤ 0.05, bold font).

As is expected, the disparity based (k-means) approach attenuates the differences in variance between groups as the objects are clustered by proximity (Fig 8B). The values of variances, however, are very similar to those seen using the body plan approach, the major differences being the split of anomalocaridids into the *Anomalocaris* and *Hurdia* clusters and the restructuring of antennulate appendages (*Sanctacaris* was excluded from the calculation of variances). The disparities of *Anomalocaris*- and *Hurdia*-like appendages, respectively, is now more similar to that of lobopodians and megacheirans+chelicerates with respect to median and total variance. They retain, however, some of the discrepancy in variance between axes, and the median disparity of *Anomalocaris*-like appendages is still greater than the disparity of other groups, because of the remarkable morphological distance between *Anomalocaris* and *Amplectobelua*.

The enlarged antennulate cluster (ANT), encompassing both stem and crown-group taxa, has now variance and median disparities both comparable to the lobopodian/opabiniid (LOP), *Hurdia* (HUR) and megacheiran/chelicerate (MEC) clusters, in contrast to the body plan-based result. The mixed cluster (MPS), on the other hand, is made up of three disparate but outlying appendages united in bearing paired spines on their inner margins, a trait of the *Anomalocaris* group. The mixed cluster (MPS) could therefore represent the anatomical convergence of three distinct frontalmost appendage types or paraphyletic relationships between these taxa at the base of different clades. Both situations can explain the inflated disparity of this group.

The disparity-based grouping sheds light on the body plan-based grouping by contrasting consistent clusters of appendages with the para- or monophyly of systematic units. The comparison notably stresses the inclusive or exclusive differences between shifts of morphology in the frontalmost appendage and shifts in the general post-frontal body plan. The very large disparity of frontal appendages in dinocaridids, for instance, results from the fact that this systematic unit contains the *Anomalocaris* and *Hurdia* clusters (ANO and HUR) for which the anatomical differences in these frontal appendages are not only highly diagnostic, but also as disparate as other body plan-based units (Fig 8). By contrast, the anatomical similarities between chelicerae and “short great appendages” led to fuse both units of related taxa, albeit with little change in the median disparity of the newly-formed group (Fig 8). In this case also, body plan and predictions based on frontalmost appendage disparity are overlapping, but the body plan-based systematic units were relatively representative, if not somewhat restrictive: the cumulative disparity of chelicerae and megacheiran “short great appendages” is now slightly greater than that of the *Hurdia* cluster (HUR), and equivalent to that the “higher” lobopodians (LOP) (Fig 8). Therefore, if, from a body plan perspective, the transition dinocaridids (DIN)-stem bivalved arthropods (BIV)-megacheirans/chelicerates (MEG/CHE) seems to be accompanied by a progressive reduction of the disparity of the frontalmost appendage, this pattern is, from the point of view of the disparity itself, incumbent upon the possibly paraphyletic nature of these body plan-based groups (as in [5, 21]) and/or the level of comparison of higher taxonomic units with each other. This encourages the use of a cladistic framework to do comparisons of disparity metrics.

The situation of the antennulate taxa seems to emphasize the stability of the appendage across body plans. Regardless of the impact of some marginal—though possibly important—morphologies (see the mixed paired-spines cluster, MPS), the antennulate s.l. cluster unites the various body plans possessing an antenna/antennule (in a likely polyphyletic fashion, i.e. stem bivalved arthropods are here allied with mandibulates, Fig 6) within a disparity equivalent to the megacheiran/chelicerate cluster (MEC). If the supposedly convergent antennule is subject to comparatively little variability in the distant clades in which it appears, then either the development or the ecology of this form of frontalmost apparatus naturally constrains its disparity.

In this case, however, the break-up of the stem bivalved arthropods by the k-means clustering conceals the critical property of this group in the morphospace, which is that it stands at the intersection of the lobopodian/opabiniid (LOP), megacheiran/chelicerate (MEC), mixed (MPS) and antennulate s.l. (ANT) clusters. There is, in particular, a triangle of bivalved taxa at this interface: *Surusicaris* (allied with MEC), *Occacaris* (allied with MPS) and *Isoxys acutangulus* (allied with the enlarged antennulate cluster, ANT). Therefore, the group designated by “stem bivalved arthropods” is not only lacking a cluster identity (contrarily to other body plans/taxonomic groups) but also has a variety of frontalmost appendages (protocerebral lobes excluded) with affinities to all other groups, and especially “short great appendages,” antennules and chelicerae.

### Significance of characters

The p-values of chi-square tests and Cramér index of character association with each axis is presented in Table 1. Influential characters are summarized by pie-charts along the first three axes in Fig 6. The most significant of these characters include the presence of secondary processes on inner spines (axis 1), the number of inner spines (significant on axis 1 but mainly shaping axis 2), the presence of paired inner spines (axis 2), the orientation of the appendage (on all axes but mainly axis 2) and the composition of the terminal cluster of discontinuously larger spines on the inner margin, or, as we call herein, inner “hand” (predominantly on axis 3). The composition of the outer “hand” dominates axis 4 (Table 1). Orientation and inner “hand” configuration are decisive in segregating the major clusters, and the number of inner spines is a more general trait providing lower-scale resolution. All the other significant traits are involved in describing anomalocaridid disparity.

The number of inner spines—not the number of podomeres—is used on axes 2 and 3 to order the appendages, mitigating the effect of large variations in segment numbers among antennulate taxa. Certainly the number of segments is a primary trait for the ordination of anomalocaridids and antennulates, but our multivariate analysis did not find its signal to be significant in discriminating frontalmost appendages on secondary axes. Values of Cramér’s V for the number of podomeres are nonetheless high on these axes, suggesting that this character remains important as a background signal beyond the first axis.

The pairing of inner spines is a character mostly impacting the second axis and dragging taxa of the mixed cluster (MPS) towards the level of the *Anomalocaris* group (ANO) (Figs 6, 7B and 7D). Although the trait itself may be convergent, the association of the multi-segmented anomalocaridid claws bearing short spinose elements with antennule-like appendages may seem consistent from a morpho-functional standpoint. The question of the anatomical resemblance between elongate anomalocaridid appendages and antennules has notably been put forward by Stein (20) based on his description of the trilobitomorph *Kiisortoqia*, but the functional or phylogenetic implications of these similarities have remained suggestive. Here, they share the same section of morphospace but the presence of secondary spines on the inner segmental outgrowths is still an important feature separating *Anomalocaris*-like appendages from the antennules of mixed cluster (MPS, axis 1, Figs 6 and 7).

Two characters that we are introducing herein are additional visible components of the morphospace axes. One is the outer “hand,” formed by a series of prominent outer spines, as in *Surusicaris* (see below), and opposed to the chelate inner “hand” of, e.g., megacheirans. The outer “hand” trait dominates the fourth axis (Table 1) to which many other characters contribute, although it is coded for only five of the taxa. In multivariate space, this character probably increases the disparity of the lobopodian/opabiniids (LOP), *Anomalocaris* (ANO) and megacheiran/chelicerate clusters (MEC, where *Surusicaris* is placed), but, as an anatomical similarity, would also reduce the distance between all these groups.

The other newly highlighted character is the rounded shape of the distalmost segment, or tip, of the appendage. Although it has remained undocumented in lobopodians and dinocaridids so far, it is the expected condition resulting from the absence or reduction of the terminal spine, and is typical of antennular appendages in general (up to a minute state). We coded its presence in bivalved taxa, and one would therefore expect the trait to be represented on the first axis of the morphospace where bivalved taxa are grouped with antennulates towards higher values. The “rounded tip” trait is indeed significant on this axis, but is also more marginally significant on axes 2 and 4, and has thus a more complex intrinsic importance.

Given the role of these traits—outer “hand” and “tip”—in describing the disparity of the frontalmost appendage, we encourage their use in further systematic and phylogenetic studies.

## Discussion

### *Surusicaris*

The body plan of *Surusicaris* is of the isoxyid type, i.e. a semi-circular carapace—excluding cardinal processes and reticulated carapace present in some species—protruding anterior and posterior extremities, large spherical eyes, a single prominent pair of frontalmost appendages articulated into a limited number of segments, and, overall, a poorly sclerotized body with, putatively, a posterior lobate tailpiece (or fan, see [22]). The type and only known specimen of *Surusicaris* is distinctive from and more informative than the *Isoxys* species as yet described. The peculiarities of *Surusicaris* revolve around four anatomical traits: three pairs of anterior unarthrodized and weakly sclerotized uniramous limbs; simple, broadly attached biramous trunk limbs; a four-segmented cephalic tagmatization and anomalocaridid-like frontalmost appendages. We discuss the implications of these characters for morphological trait distribution and homologies within stem panarthropods.

#### Anterior limbs

The post-frontal limbs of *Surusicaris*, and especially the three anterior uniramous pairs (Figs 1A, 1C, 1D, 2A, 2B, 3A and 4A) lack arthrodization in the classical sense (i.e. clearly delimited segments articulated through arthrodial membranes), and the degree of sclerotization appears especially weak in the compact, extremely short “segments” of the post-frontal uniramous limbs. *Aysheaia* Walcott (Fig 4C) and a stouter form, *Hadranax augustus* Budd and Peel, 1998 (Fig 4D), have lobopods whose annulation pattern is preserved similarly to the cuticular structure of the anterior limbs of *Surusicaris*. Interestingly, the Chinese lobopodian *Diania cactiformis* Liu et al. [78] bears limbs whose aspect is in fact more segmental (Fig 4B) than in *Surusicaris* (Fig 4A), possibly illustrating a relatively homoplastic state of limb sclerotization between derived lobopodian and basal arthropods.

Taphonomic deformation can sometimes reshape an arthrodized leg into a more compact and rounded appendage, such as seen in, e.g., *Canadaspis* Walcott (Fig 4F). Based on a redescription of *Diania*, Ma et al. [79] discussed the morphological and taphonomic criteria necessary to make a distinction between annulation and segmentation, and their principal argument relies on the consistency of marginal outlines. In *Surusicaris*, indeed, annulations vary greatly in shape, their preservation quality is inconsistent, and, more importantly, their number possibly differs between limbs. These characteristics can be unambiguously opposed to the preservation of loose segments’ shape in “poorly” sclerotized limbs, in which the general elongated habitus usually remain characteristic of arthrodization (see, e.g., in *Odaraia* Walcott and *Chengjiangocaris* Yang et al., Fig 4E and 4G herein). Moreover, a comparison with the segments of the clearly arthrodized frontalmost appendages strongly supports that these observations are not taphonomic artifacts. If not annulated in the lobopodian sense, the three pairs of anterior limbs of *Surusicaris* are at most weakly sclerotized and, in the context of stem bivalved arthropods, certainly represent a plesiomorphic condition.

### Biramous trunk limbs

The trunk limbs of *Isoxys* have been interpreted as “typical” stem arthropod limbs, that is, composed of a leg-like endopod and a paddle-like setose exopod attached basally [32]. Interestingly, however, what has been tentatively considered as the “endopod” by García-Bellido et al. ([32], Fig 2C1) in more poorly preserved material is strikingly reminiscent of the darker inner traces observed in *Surusicaris* (Figs 1, 2, 3, 4A, 5A and 5E–5G), traces similarly associated with the gut. It appears to us that the analysis of the limbs of *Isoxys* might have suffered from their equivocal preservation, and that the broadly attached endopod and exopod displayed by *Surusicaris* could shed light on their real nature. The species *Isoxys volucris* Williams [80] from Sirius Passet might corroborate the view of García-Bellido et al., but the poorer preservation of the material makes any comparative criticism difficult.

The carbonaceous traces in the limbs of *Surusicaris* are preserved ventral to the gut (Figs 1A, 2A, 2B and 3A), corroborating an internal position. Although they clearly adjoin the central digestive system visible in the posterior part of the body (Figs 1A, 2A, 2B, 3A and 4A), the reflective strips are preserved with a much denser carbonaceous film throughout, emphasizing the distinct identity of this tissue from other limb or gut tissues. Those strips seem complemented by a larger, darker spot at the base of the limb (Figs 2C–2E, 5A, 5E and 5F), and extend deeply within the anterior legs and exopods of the biramous trunk limbs. In the trunk limbs, they further divide into three branches, the medial branch thinner than the ones on either side (Figs 1A, 2, 3B and 5A–5G). The tissue between those channels, as in other parts of the body other than the gut and the eyes, was in *Surusicaris* ultimately mineralized into an Fe-Mg compound (Figs 2E and 5F), a characteristic common in other arthropods of the Marble Canyon deposit currently under investigation.

Gut-related lateral structures that are segmentally repeated in the trunk have been described in early Palaeozoic arthropods with soft body preservation, especially megacheirans and trilobitomorphs, and are generally referred to as “mid-gut glands” [81] and “caeca/diverticula” (e.g. [82–86]). In the case of trilobitomorphs, they can complement more or less ramified caeca occupying or merging with the cephalon, although such caeca are most commonly found in trilobites to be anastomosed vascular systems rather than alimentary diverticula [83–85]. Similarly to *Surusicaris*, the medial duct in certain naraoiids [85] is adjoined by branching diverticula that are sometimes preserved as volumes of sediments or calcium phosphate, or as distinctly dense carbonaceous strips, and which show the same secondary tridental branching pattern (Vannier and Chen [85], Figs 1 and 3; this paper, Fig 4C). To explain the occasional taphonomic differences, Vannier and Chen stressed the peculiarities of enzyme synthesis and food storage functions that likely took place in the caeca, in contrast to the functions occurring in the intestine proper [85].

Hepatopancreatic caeca can be fused as rami, which are then referred to in crustaceans as the hepatopancreas, or they may be fused serially and segmentally repeated, a condition present in early arthropods and that is of interest to us here. Crown-group arthropods such as scorpions [87], copepods [88] and remipedes [89] possess paired hepatopancreatic caeca in trunk segments, suggesting that this anatomical characteristic is still widespread from a phylogenetic perspective, even if it is rare and certainly homoplastic. The lateral extension of caeca in naraoiids and extant taxa appears to be limited, although blind lobate extensions have been reported in the legs of spiders ([90], this paper, Fig 5B). In Araneae, however, the disposition of caeca is anatomically constrained to be ramified and not strictly branched off the intestine because of the presence of a sucking stomach.

In *Surusicaris*, the distinction between “cephalic” and trunk diverticula is limited to the absence of secondary branching in the anterior uniramous legs, and the caecal system reproduces the same serialized pattern throughout its length, since the “head” itself is only roughly tagmatized. The caeca are stretched to the distal end of the limbs—a configuration not known to us in other arthropods—although this condition may be an exaggeration of a situation similar to, e.g., spiders (Fig 5B).

However, the branched patterns of the trunk limbs seem to be constrained within the area of the exopods, as if the structures were associated. If this were the case, it may then be analogous to the phyllopodous limbs of some extant taxa in which the exopod is framed by a trident of haemolymph channels. An example of such a limb has already been provided by Shu et al. [18] in the hypothetical condition of *Kunmingella*, based on the thoracopods of the leptostracan (Crustacea:Malacostraca) *Dahlella caldariensis* Hessler (Fig 5D). They are called turgor appendages, the shape of which is obtained thanks to the inflation resulting from haemolymph pressure [91]. A more general interpretation for the circumintestinal traces seen in Burgess Shale fossils has been that of “body cavities,” as proposed by Whittington [92] or Budd and Daley [93]. Such cavities are present in the lobopods of onychophorans and arachnids (Dunlop and Edgecombe, pers. comm. 2013), although, in these cases, they do not form the coherent central structure we observe in fossils. These cavities are filled with haemolymph, hydrostatically maintaining the shape of the limb in a similar way to a turgor appendage. Accordingly, either the cavity walls or presumably organic-rich fluid fillings would be preserved as kerogen traces, as is otherwise standard for Burgess Shale-Type preservation [73, 81, 94]. Cases of outstanding density of these traces compared to surrounding tissues can barely be explained by the preservation of cavity walls alone, but in certain circumstances presumed internal decay-fluids usually called “dark stains” are known in fossils of the Burgess Shale to be especially conspicuous [95]. Such stains, however, usually only visible along the margins of body parts, are highly variable in shape and extent, and have diffuse boundaries. In *Surusicaris*, the distinct outline of the “stains” and their finely preserved connections with the intestine (Figs 1A, 2A, 2B and 3A) points to afferent tissues rather than to the infilling of an internal body cavity by fluids.

**Fig 9:**
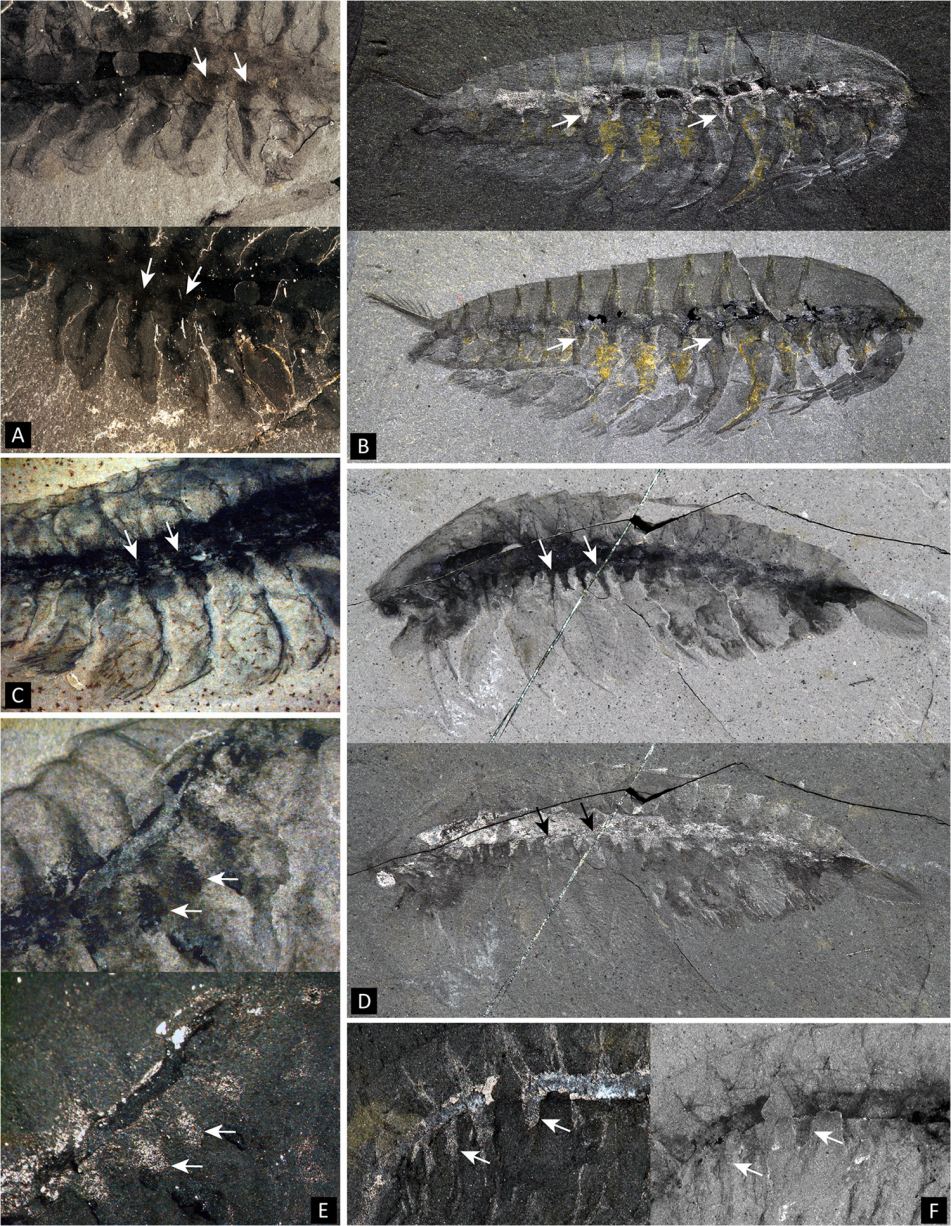
Internal sub-triangular structures at the base of trunk limbs in several Cambrian panarthropods. Note that they are part of a broader axial region (white arrows point to selected examples). **A.** Part (above) and counterpart (below) of *Opabinia regalis* Walcott USNM 155059b. **B.** *Leanchoilia persephone* Simonetta ROM 57666. **C.** *Alalcomenaeus cambricus* Simonetta ROM 45613. Here, the triangular bases seem to be somewhat prolonged onto the anterior margin of the exopods, similarly to what is often observed along the lobes of *Opabinia*. **D.** *Alalcomenaeus cambricus* ROM 53352. **E.** *Alalcomenaeus* sp. (ROM 62968, Marble Canyon deposit). **F.** *Molaria spinifera* Walcott USNM 57688 (lectotype). A, B (bottom), C, D (top), E (top), F (right) images taken under cross-polarized light, all other images taken under water using direct light. Scale numbers in mm.

A range of Burgess Shale panarthropods, such as *Opabina* Walcott, *Molaria* Walcott, *Alalcomenaeus* and *Leanchoilia* exhibit ventro-lateral projections seemingly related to the intestinal tract and more or less invading the base of the legs (Fig 9). This aspect of the anatomy of those taxa emphasizes the existence of a broad axial band to which the “caeca” appear afferent, and distinct from the calcium-phosphate infill seen in the intestinal cavity *per se*. The triangular strips projecting into the bases of the limbs are distinct from the “mid-gut” glands *sensu* Butterfield [81]; Budd and Daley [93] argued and illustrated the taphonomic distinction between the mid-gut glands and the triangular strips at the base of the lobes in *Opabinia* (Fig 9A), and this is also the case in e.g. leanchoiliids (Fig 9B–9E) or artiopods (Fig 9F) in which the paired three-dimensional pellets located dorsally of the alimentary canal neatly differ from the ventral tonguelets. Whether the sub-intestinal tonguelets could represent additional digestive structures, and whether there are homologous to the internal limb structures of *Surusicaris* and perhaps certain artiopods remains to be determined.

#### Frontalmost appendage

The frontalmost appendage of *Surusicaris* is oriented upward. It is made up of five segments and ends distally in four (three+one) elongated spinose elements (Figs 1B–1D and 3A). This combination of traits has so far been considered diagnostic of the “short great appendage” of megacheirans [11], and in the absence of terminal flagella, specifically of the yohoiid type (e.g. 4, 11).

The additional spinose processes on the inner margin of the medial segments of the appendage (Figs 1B–1D and 3A), however, could be reminiscent of an anomalocaridid condition [49, 96, 97], but are more generally, a plesiomorphic state seen in the various arthrodized appendages of a number of stem arthropods (including cephalic and trunk legs, see e.g. *Canadaspis perfecta* Walcott in (43)). To some extent, a conspicuously spinose inner margin can also be found in the post-cheliceral appendages of xenopods and arachnomorphs [98, 99].

**Fig 10:**
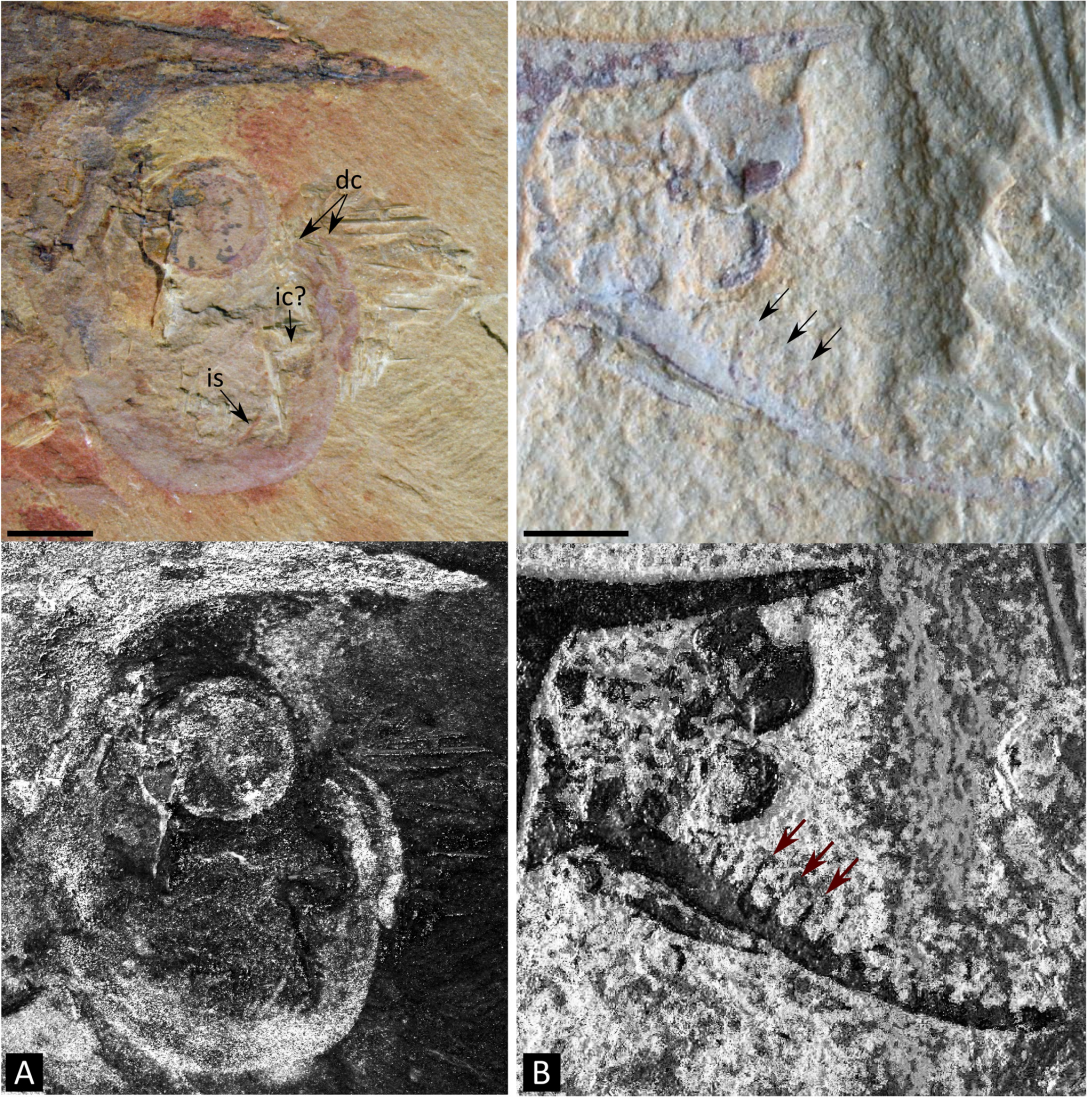
Possible examples of larger disparity in the frontalmost appendage of *Isoxys* types from the Chengjiang Lagerstätte. **A.** Unpublished specimen (CAL04). The appendage shows similarities with *Surusicaris elegans* gen. et sp. nov. in being upward-directed, few-segmented and “raptorial,” although the distal claw is possibly made of parallel spines. **B.** HSX08 (as in Vannier et al. [13]). The appendage is here multi-segmented and antenna-like. Top images taken under cross-polarized light, bottom images with enhanced contrasts. (original photographs courtesy Jean Vannier). **Abbr.** dc: distal claws; e: eye(s); ic: inner claw; is: inner spine. Scale numbers in mm.

A spinose cuticular outgrowth of the segments’ inner margin is also present in *Isoxys*. In *I*. *acutangulus*, a single or two-segmented peduncle is followed by three more developed segments, of which the first two bear short and large spines across specimens, while the last markedly ends in a slender ‘tip.’ As Vannier et al. [13] propose, this distal element could very well be a distinct, fourth terminal segment. Structurally, this frontalmost appendage therefore could roughly correspond to the basic “short great appendage,” with the four distal segments being the “multi-chela” of occacaridids and megacheirans [13]. The one-segmented base—two-segmented in megacheirans [11]—constitutes nevertheless a notable difference. In addition, there are indications of interspecific variation within or in the vicinity of this genus: spines are vestigial in e.g. *I*. *acutangulus* Walcott, but much thinner and longer in *I*. *volucris* as well as in a single unnamed Chinese specimen published by Vannier et al. ([13], Fig 3J; see also Fig 10B herein). It thus seems that the disparity of *Isoxys* species, or genera under Isoxyidae, is underestimated and cannot easily be reduced to the morphology of the “short great appendages.”

There is, in fact, a fundamental structural difference in the terminal portion between the frontalmost appendage of *Surusicaris* and that of megacheiran arthropods. In megacheiran arthropods, the distal portion of the appendage is composed of a set of spinose outgrowths on the internal, or inner margin of successive distal segments [4, 11], while in *Surusicaris*, the insertion of the two posteriormost distal spinose processes is distinctly external, while the anteriormost is apparently sub-terminal (Figs 1B–1D and 3A). This condition in *Surusicaris* is more similar to that of certain anomalocaridids, e.g. *Anomalocaris* and *Amplectobelua*, in which the segments, sometimes adorned with both inner and outer spines along a substantial part of the appendage, are distally produced into longer outer spines (e.g. [49, 97, 100]). It is impossible to determine in the holotype of *Surusicaris* whether each terminal spine also corresponds with an individual segment, but the insertion of the spines on the outer margins of the segments, as in anomalocaridids, suggests this could be the case. The similarity of the distal portions also suggests a homology of the proximal inner outgrowths with those of anomalocaridids but, likewise, too little is known from the holotype to compare them in detail. Nonetheless, the base of the proximal spinose outgrowths (Figs 1B–1D and 3A) are clearly medial and there appears to be only one per segment; additional spinose tips are those of the other flanking appendage (Fig 1B and 1C). Given the position of dinocaridids as sister group to the euarthropods [21, 77, 101], this observation both suggests a plesiomorphic condition of the frontalmost appendage of *Surusicaris*, and corroborates the extended disparity of frontalmost appendages within the Isoxyidae, of which there are probably other, undescribed examples (Fig 10). This finding emphasizes the pivotal role of isoxyids and their relatives in the transition towards the euarthropod morphology [22], the indirect or direct nature of this role in the case of the frontal appendage being conditional upon the topological identity of this apparatus between isoxyids, dinocaridids and other arthropods.

#### Anterior tagmatization

The frontalmost appendages of *Surusicaris* are followed by three uniramous pairs of poorly sclerotized limbs that are distinctly different from the morphology of the biramous trunk limbs (Figs 1A, 1C, 1D, 4A and 5A). In general, the differentiation of immediately post-frontal appendages in arthropods is used to define the “head” section, or cephalon [19], regardless of whether the cephalic segments correspond to the overlap of a fused anteriormost tergite or are decoupled with this overlap (as in fuxianhuiids [6] or in general bivalved arthropods). We here construe that the eyes, the frontalmost appendages and the following three uniramous limb pairs of *Surusicaris* form one tagma—the cephalon. Although they are particularly conspicuous in *Surusicaris*, whether such three pairs of uniramous, poorly sclerotized legs exist in *Isoxys* cannot at present be ascertained. Similar legs have been suggested in *Kunmingella* [18], but the clarity of the evidence may be disputed.

Following a parsimonious view, the cephalon of *Surusicaris* is therefore composed of five somites—one ocular, one “antennal”, and three pedial—a configuration argued to be the a synapomorphy of Euarthropoda in the sense of Walossek [17] and Maas and Walossek [102], though, contrary to their conception (largely inspired by the trilobitomorph body plan), the three post-“antennal” pairs are, in this case, uniramous.

The phylogenetic position of *Surusicaris* is still to be tested in light of its whole diagnosis in order to infer the significance of the “head” character. All together, the five-somited head, the unarthrodized anterior uniramous limbs and the dinocaridid-like frontalmost appendage are strongly suggestive of a basal position relative to both euarthropods and “stem bivalved” arthropods. The condition of a five-somite head, however, seems in stark contrast with the pattern of one or two pairs of close anteriormost differentiated appendages some authors see to be common between e.g. “stem bivalved” arthropods and fuxianhuiids [5, 21]. According, e.g., to the topology in Legg et al. [21], the condition of the *Surusicaris* cephalon would be derived, but such a scenario then conflicts with the lack of limb arthrodization and the poor differentiation of endopod and exopod, as well as with the affinity of the frontalmost appendage with dinocaridids. Some of these characters may be subject to homoplasy, but their simultaneous presence in *Surusicaris* may be explained by a different scenario of arthropod evolution than the ones currently proposed, or otherwise by specific macroevolutionary processes such as parallelism and morphological plasticity. It may be interesting to note that putative “head” appendages have also been discussed in the case of *Hurdia* [77, 96] and *Anomalocaris* [97], which could illuminate the origin of the anterior differentiation in *Surusicaris* and support either a plesiomorphic placement of this taxon or the peculiarism/need of reinterpretation of the arthropods with so-called “two-segmented” cephalons.

### Disparity and evolutionary scenarios

**Fig 11:**
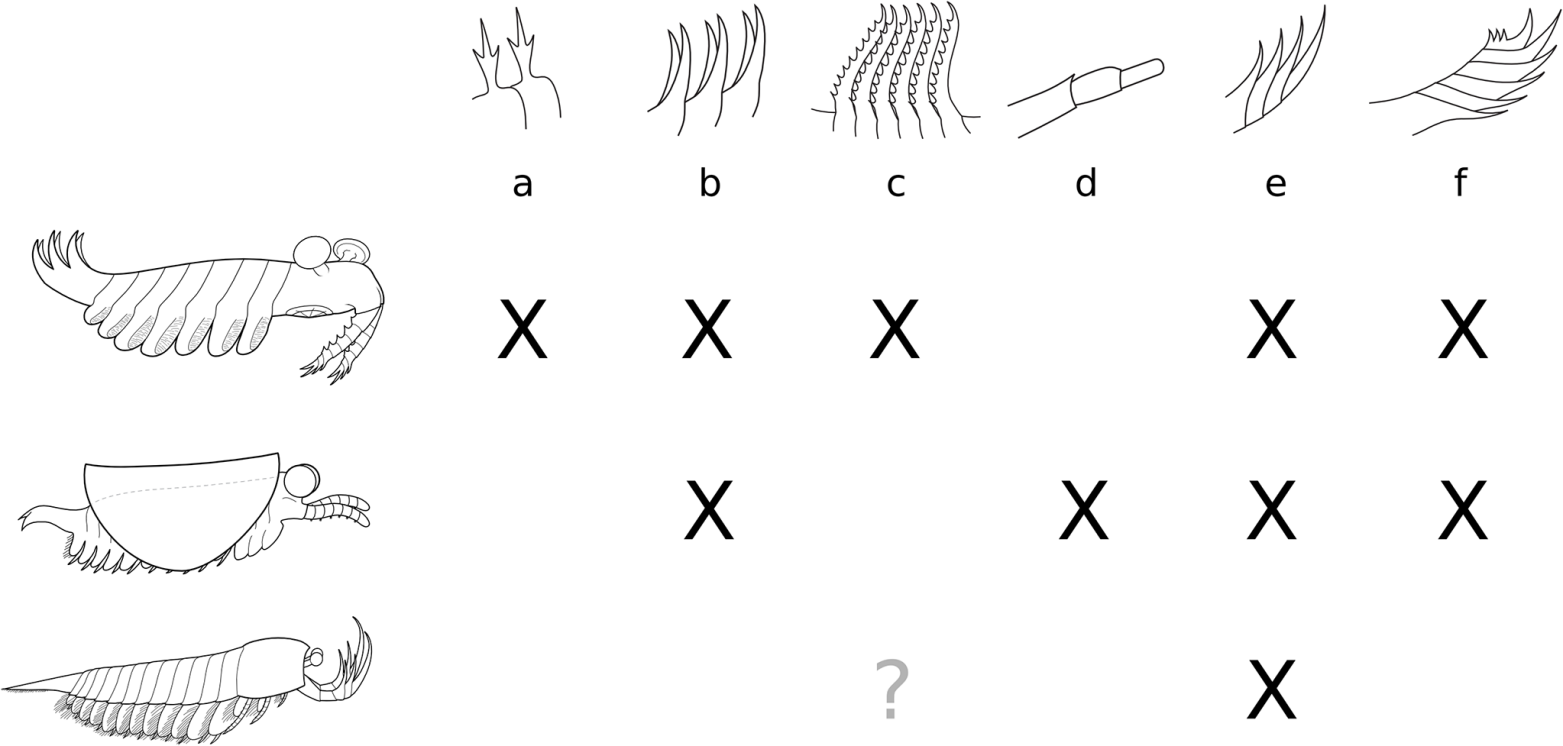
Distribution of important traits of the frontalmost appendage among the three main stem body plans considered herein (from top to bottom): dinocaridid, stem ‘bivalved’ and megacheiran. The question mark (3^rd^ row, column c) refers to the problematic condition of *Sanctacaris* Briggs and Collins. **Characters:** a: secondary processes on inner spines; b: double row of inner spines; c: elongate, slender inner outgrowths; d: rounded undifferentiated tip; e: composition of distal “hand” formed by the differentiation of inner spines; f: composition of distal “hand” formed by the differentiation of outer spines.

A summary of the known correspondences between body plans and the most influential morphospace characters of the frontalmost appendage is provided in Fig 11. In light of the remarks above, it can be seen that the characters driving the frontalmost disparity are decoupled from the rest of the body plan between anomalocaridids and stem bivalved arthropods, and that these two groups dominate the disparity of this appendage.

Drivers of disparity in anomalocaridids are related to the variety of available prey [103, 104], but also, probably, to radically different feeding strategies. Variation in feeding strategies has recently been highlighted by the suspension-feeding reinterpretation of *Tamisiocaris* frontal appendages [76], and, more generally, by a criticism of the preconceptions about feeding in anomalocaridids [105, 106].

For supporters of a proto- to deutocerebral topological transition along the stem Arthropoda, the structural evidence either supports a change within or after bivalved taxa, or a high degree of convergence of the deutocerebral apparatus at the time of its development in bivalved taxa. Interestingly, the former hypothesis could infer a higher disparity for the protocerebral appendage compared to the deutocerebral one, whereas the latter supposes that the deutocerebral experienced a short burst of disparity before being quickly constrained. In either case, insight into this transition can only be given by carefully examining the relationships between bivalved morphotypes.

The odd resolution of bivalved taxa in the morphospace represents a morphological “anomaly” possibly in accordance with the hypothesis of topological shift of the frontalmost appendage, as one would expect the reduction of an existing appendage and the development of a new one to involve large morphological changes. From a more conservative point of view, this phase, as shown by the organization of the morphospace, would correspond to a wholesale modification of the deutocerebral appendage into antennular, “short great appendage” or chelicerate-like morphologies. The view that euarthropod clades branch from different “stem bivalved” groups has, however, never been supported [5, 21, 107, 108]. This suggests the decoupling of frontalmost appendage disparity from the rest of the body and points to a striking degree of plasticity and parallelism in these forms.

Another scenario would be that the emergence of the main frontal appendage morphologies predates in fact their diversification within the “bivalved” group. In theory, on the same structural basis that led Chen et al. [4] and Haug et al. [11] to propose a continuous link between anomalocaridid appendages—and more precisely, the *Hurdia* type—and those of megacheirans, a similar evolutionary scenario could be proposed between the multi-segmented frontal appendage of the *Anomalocaris* type and antennule-like appendages (e.g. [20]). As a trivial consequence, however, these scenarios conflict on the origin of key euarthropod synapomorphies, or assume, given our current understanding of anomalocaridid morphology [49], unrealistic pathways of convergences involving critical characters such as arthrodization and biramicity of axial limbs. Although the anomalocaridids show a morphological dichotomy of frontalmost appendages in terms of disparity measurements (Figs 6, 7 and 8), there is no current support for an “anomalocaridid-centered” radiation of arthropods in light of the whole body plan (not even, in fact, structurally) according to which a polyphyletic Arthropoda would emerge from different anomalocaridid lineages. What those results rather demonstrate is the distinct structural identity of anomalocaridid frontal appendages overall but also the strong functional identity of subgroups of anomalocaridid appendages (clades? See e.g. [76]). As a matter of fact, the specialization of anomalocaridid frontal appendages, not only as predators—with respect to their coevolution with specific prey items,—but in feeding strategies overall [76], involved the plasticity of a unique combination of constituting traits contrasting with the diversity based on shape or secondary structural features in euarthropods.

In the context of topological differences between appendages, it may be objected that characters not applicable to all taxa may account for a degree of disparity among homologous appendages not captured here (e.g. number of teeth on the inner margin of a cheliceral claw)—implying that plasticity may affect different traits in different homologous structures. In addition to the fact that, here, numerous finer traits have also not been coded for e.g. dinocaridids, one would expect the coding of autapomorphic characters to change within-group distances and increase the distance between groups (if the characters influence any significant axis), but not the total amount of morphospace occupied. This would require further testing, although it seems reasonable that only a measure of disparity based on segmental morphology—as is the case herein—can encompass variations in all taxa.

It may be that protocerebral and deutocerebral appendages are not equally plastic/variable, or that their variation would not involve the same type of secondary traits. However, if we strictly compare differences in disparity between dinocaridids/“stem bivalved” arthropods and other arthropods, this does not explain the observed pattern of *decreased* disparity. Certainly this could be integrated within the general scheme of increased evolutionary constraints in the early history of large clades [26, 29, 109–112], and the integration of genetic/developmental and ecological factors—especially with respect to the “Cambrian Explosion” [27, 28, 113–118]. A more direct cause in this very case would simply be the concomitant selection of differentiated appendages: as functions are taken over by additional limbs (palps, maxillae, etc.), the frontalmost appendage becomes involved in less disparate morphological changes, and more in refinements of the selected function of the group (antennula, chelicera).

Of course, the relatively low disparity of “short great appendages,” in the absence of clear posterior cephalic appendage differentiation in these animals is then difficult to justify with this hypothesis alone. However, as it has been stressed above, these general observations of patterns between paraphyletic and monophyletic clusters are suggestive at best. We have seen notably that the pattern of disparity is much stable when taxonomic units are broken down (or fused) into groups with stronger structural affinities. As it appears that some of these groups may also form clades (ANO, HUR, ANT (as Artiopoda+Crustacea) and MEC?), this suggests that monophyletic units with a certain type of appendage may be affected by a limited disparity distinct from the overall pattern. To clarify this, it is necessary to integrate a node-by-node mapping in the study of disparity, and to extend the analysis to the rest of the body plan.

## Conclusions on Disparity

The Cambrian radiation has more dimensions than a branching cladogram. Put forward notably by Gould [24, 113], the application of alternative methods such as morphospaces to aid in understanding the evolutionary significance of the outstanding disparity displayed by Burgess Shale-type communities has had limited resonance so far, although they account for the explanatory patterns of body plan evolution [25, 26, 29, 111, 113, 119–121].

Our morphospace has implications both dependent on and independent of phylogenetic relationships. In light of existing cladistic results, the ordination of disparity across frontalmost appendages is mostly consistent with cladification, but the comparable values of morphological variances do not correspond to the taxonomic levels the systematic classification has recognized so far. From the point of view of systematics and body plans, there is a trend of decreasing disparity of the frontalmost appendage; from the point of view of this appendage alone, taxa sharing a relatively common general morphology of the frontalmost appendage tend to be comparatively dispersed. What this means is that, at different levels of body plan evolution in arthropods, successful morphologies of the frontalmost appendage are characterized by similar degrees of morphological constraint relative to the overall morphospace. In other words, the turnover of structural categories of frontalmost appendages is higher than the turnover of the body plan as a whole. At the same time, however, we have seen that characters associated with highest disparity (e.g. size of inner and outer “hands,” presence of paired spines, etc.) were reaching across dinocaridid and bivalved bodyplans (Fig 11), suggesting another, superimposed level of control of disparity.

One “group,” difficult to recognize using descriptions of the frontalmost appendage alone, does stand out by its extended and central disparity—the one composed of the so-called (stem) “bivalved arthropods,” including *Surusicaris*. The morphological affinities of these taxa with dinocaridid frontal appendages, megacheiran “short great appendages” and antennules are consistent with the claim of a pivotal position along the dinocaridid/euarthropod transition. A hypothesis of deutocerebral continuity from at least dinocaridids onwards would illustrate the exceptional parallelism in these taxa and the fact that a burst of morphological variation preceded the co-optation of functional types in euarthropods; an alternative hypothesis of proto- to deutocerebral topological transition among bivalved taxa would also point out the fact that this burst of disparity is associated with the emergence of the deutocerebral appendage. Importantly however, a node-by-node dissection of the evolution of disparity across these taxa is necessary to know whether this burst corresponded to a single evolutionary event, to an event relatively isolated from the main euarthropod lineage or to a number of scattered adaptations inflating the overall disparity value when put together. Indeed the anatomy of many “stem bivalved” forms—of the head in particular—remains unclear to a large extent, so that their phylogenetic placement and the extent of polyphyly for these taxa are still issues to be addressed. Characters that in bradoriids for instance could be interpreted as the generalized plesiomorphic antennulate form in “stem bivalved” arthropods, such as a short and stout antennule with inner margin setation, were also recognized as characterizing the plesiomorphic crustacean condition [18]—as e.g. in phosphatocopines [122] or, possibly by reversal, in cephalocarids [123]. We herein integrated this observation as part of the “stem bivalved” parallelism, but this also strongly lays stress upon the “bivalved” body plan being mostly an association between a possibly very plastic bivalved condition [124] and an assemblage of divergent/poorly understood anatomies. The question to answer now is therefore if the evolution of a bivalved carapace is simply much more prone to convergence than the frontalmost appendage, and if that means that the recognition of spread-out disparity of “bivalved taxa” in the morphospace is at least partially an artifact.

The disparity pattern of the frontalmost appendage is of course not entirely self-explanatory. It would be expected that the delegation of function to increasingly differentiated posterior appendages led to changes in the constraints affecting the frontalmost appendage, and ultimately changes in range or type of disparity. This hypothesis is to some extent verified by our results, though a more direct and quantified comparison is needed to fully test it.

Complicating this issue is the fact that frontalmost appendages and more posterior ones might share pieces of the developmental toolkit. It is likely that in the evolution of arthropods there have been degrees of antero-posterior serial differentiations [125], one of them possibly being the co-optation of the pattern of the segmented frontalmost appendage in more posterior limbs. Besides the possible structural similarities between proto- and deutocerebral appendages highlighted herein, trunk limbs such as those in *Canadaspis* [43], for instance, are built on a model intriguingly close to that of the anomalocaridid frontal appendage, with long inner spines and reduced terminal ones, including a number of outgrowths on the outer margin. Although plesiomorphic characters are retained extensively throughout stem arthropods, a parallel comparison between frontalmost appendages and posterior limbs (e.g. in the same morphospace) might tell us more about the constraints at work during the canalization of early arthropod body plans.

To summarize,

-Surusicaris gen. et sp. nov. is an isoxyid whose frontalmost appendage bears structural affinities with those of anomalocaridids; the animal also is characterized by unarthrodized limbs, which in the trunk are biramous but with largely fused branches—an a priori very basal condition of limb morphology;
-Surusicaris has a “proto” head composed of a pair of large eyes, a frontalmost appendage and three pairs of uniramous limb pairs. This would ally the fossil with euarthropods sensu Walossek [17] that have a four-segmented head; as a corollary, the isoxyid frontalmost appendage would be deutocerebral; the uniramicity itself may equally be ancestral or derived, and in the latter case likely convergent;
-Phylogenetic analyses should discriminate between heritable signal and convergence among this association of plesiomorphic and derived features, but Surusicaris and the other isoxyids certainly highlights unusual plasticity potentials in basal arthropod. This also stresses the issue of our poor knowledge of head configuration in the entire group designated as “stem bivalved” arthropods;
-In the strict sense of Gould [113], the frontalmost appendage of “stem” arthropods is more disparate than that of “crown” ones. If one looks at body plans instead, the disparity decreases along the general phylogenetic succession dinocaridids-“stem bivalved taxa”-megacheiran/artiopods-crown;
-The inherent clustering signal of the morphospace suggests that such readings are very reductive of the evolutionary pattern, and that a clade-based comparison may be much more appropriate. Overall, body plans can be split or combined to constitute disparity-based groups with more comparable values of variance and, to some extent, additional phylogenetic significance;
-The general exception is the “stem bivalved” group. They are scattered amongst different clusters but within a sector located at the interface between major frontalmost appendage types. This interestingly could correspond to a burst of disparity prior to the diversification of various functional types in more derived clades, and even, given a hypothesis of proto- to deutocerebral transition, a higher disparity in the very early evolution of the deutocerebral apparatus;
-The significance of “stem bivalved” taxa in the early evolution of arthropod disparity (here, in the case of the frontalmost appendage) needs to be further evaluated in light of more anatomical evidence—especially regarding head configuration—and of an assessment of the impact of polyphyly on generating such a result;

Decoupling, i.e. the relatively independent evolutionary trajectory of body parts, is an important indicator of evolvability at the macroevolutionary level. Our results suggest several imbricate grades of decoupling in stem arthropods: [1] first rank morphology of frontalmost appendage matches body plan well; [2] second rank morphology of frontalmost appendage is decoupled from body plan, leading to tight within-body plan clusters; [3] specific traits of frontalmost appendage transcends body plans on the contrary, meaning that [1] and [2] are not dependent on individual traits but on trait combinations.

## Supporting Information

S1 Comment Overview of the “great appendage” disparity. (DOC)

S1 Dataset Dataset and description of characters used in this study. (DOC)

S1 Fig Complementary analytical tests on the morphospace. **A.** Scree plot of the PCoA analysis showing that most of the variance is explained by the first four axes. **B.** Procedural k-means partitioning on the first four axes of the PCoA set for 3 to 7 groups. The Calinski criterion found an optimum at 6 groups.
(TIF)

S2 Fig *Sanctacaris* Briggs and Collins, a stem-group arthropod with “great appendage”-like anterior limbs. *Sanctacaris uncata*, part of the holotype (ROM 43502). **A.** Close-up of the head. Note the presence of at least one differentiated biramous appendage behind the antenniform appendage (see “rcex” and “lcba”) as well as the secondary spinose outgrowths of tridental shape on the frontalmost “legs,” reminiscent of *Anomalocaris* (e.g. Daley and Edgecombe [97]). Additional preparation reveals thick endopods associated with the trunk segments. **B.** Close-up of the left pleura in A. The biramous cephalic appendage visible here is composed of an endopod (“men”) whose shape is highly differentiated into a rod bearing distal setae. Close resemblances can be found among the maxillae or maxillules of certain extant crustaceans (e.g. Cephalocarida, see Sanders [123]). **Abbr.** arfa?: antennular ramus of the frontal appendage?; bsh: broken spinose hand; cex?: cephalic exopod?; ex: exopod; if: internal ‘filament;’ la: left appendage; lcba: left cephalic biramous appendage; lcex?: left cephalic exopod?; ltex: left trunk exopod; mra: margin of right appendage; men: maxilla-like endopod; ra: right appendage; rcex: right cephalic exopod; rten*x*: right trunk endopod (1–2); sbfa?: secondary branch of frontal appendage?; sh: spinose hand; ten: trunk endopod. Scale numbers in mm.
(TIF)
