# Supplementary material for: Cephalic and Limb Anatomy of a New Isoxyid from the Burgess Shale and the Role of “Stem Bivalved Arthropods” in the Disparity of the Frontalmost Appendage
Source: PLoS One. 2015 Jun 3;10(6):e0124979. doi: 10.1371/journal.pone.0124979 (PMC4454494; doi:10.1371/journal.pone.0124979)
Supplement: S1 Comment — (DOC) [file pone.0124979.s001.doc]

**Disparity and definition of the “great appendages”**

Apart from the uncertainties regarding its homology with crown-group frontal apparatuses, the very elusive terminology of “great appendage” pertains because the inherited anatomical lexicon of “antenna” and “chelicera” is either too broadly or too narrowly applicable for structures that could be defined as none of these terms, or both at the same time. A great deal on this matter has already been covered in detail by Chen et al. [1] and Haug et al. [2], and our purpose is only to clarify and expand their studies from a purely anatomical, and possibly analogical, point of view.

The “great appendage,” in its leanchoiliid and yohoiid versions (= “short great appendage”), derives from an arthrodized limb whose distal spines have differentiated altogether and can act as a raptorial complex, but may have been more ecologically diverse [3]. Differentiation of other spines on the inner margin is decoupled. An antennal analogy in leanchoiliids is also evident, the difference with the ‘mandibulate’ antenna being that the latter is at most bifid (in crustaceans). Exceptions can be found in branchiopods, in which the second antenna of anostracans (with mating or feeding functions, see e.g. [4]) can be dramatically ramified (e.g. *Chirocephalus*), as well as in the modified first thoracopod of notostracans, in which a third rami diverges proximally. The evolutionary/functional significance of those analogies (under the conception that “great appendages” do correspond to A1=antennule=first antenna) is not clear.

The “short great appendage” would thus be characterised by its multifid (also more partially called “multi-chelate,” in reference to the grasping function and functionality of individual spines) termination in which rami derive from spinose cuticular projections of distal segments. Its base abuts the mouth anteriorly – when a hypostome is present, the appendage is probably attached through the postero-lateral margins of the plate [5,6], although the consistency of the latter trait still needs to be thoroughly tested.

Including the isoxyid type and, *a fortiori*, the lobopodian and anomalocaridid types, proves more difficult. The above terminology suffers from an imprecision – epitomised by *Surusicaris* and anomalocaridids, as well as possibly by some lobopodians – regarding the different states of development and origins of the ‘multifid’ tip, viz. that spinose cuticular outgrowths can originate either from the outer margin of the podomeres, or from the inner margin. It consequently renders the characterisation of the ‘bare’ *Isoxys* frontal appendage ambiguous. Two critical characters in this regard can be used to relate isoxyids, *in part*, to the ‘terminal-inner-finger-bearing great appendages,’ while at the same time emphasising the segregation between this newly formed taxon and anomalocaridids/more basal panarthropods. We need to keep in mind however that these two characters are, as we noted earlier, certainly variable within isoxyids themselves.

The first one is the number and relative size of podomeres. There is an overall discrepancy between the rather well constrained base structure of the typical megacheiran “short great appendage” (two proximal articulating segments and four distal more or less spinose articles) and the variation seen in the frontal appendages of lobopodians/dinocaridids (and, in all likelihood, as mentioned above, certain *Isoxys* morphotypes.). The *Hurdia* type (to which we can certainly add *Schinderhannes* Kühl et al. [7], *Parapeytoia* Hou, Bergstrom and Ahlberg [8] and maybe *Cassubia* Dzik and Lendzion [9]) can represent a structural intermediary between the anomolacaridid appendages and the ‘short great appendages’ [2,7,10]. While the podomere number helps differentiate “great appendages” from polysegmented antennae, this character is more equivocal when considering the simpler antennules of antennulate stem bivalved arthropods or certain crustaceans (e.g. Cephalocarida Sanders [11]).

Within the panchelicerates, a multi-segmented proximal portion is nevertheless a plesiomorphic form of the megacheiran appendage, likely to exceed the two currently diagnostic proximal podomeres [1,2], hypothetically further reduced to one and ultimately, in the most derived chelicerae, reduced entirely. Sharma et al. [12] have recently identified the role of the gene *dachshund* (*dac*) in the segmental identity of the proximal cheliceral article, showing that a loss of expression of *dac* is probably at the origin of the two-segmented chelicera. Such a shift in gene expression might have happened repetedly along with the shortening of this appendage, and even originally within dinocaridids. Xiphosuran-like early chelicerates such as *Offacolus* Sutton et al. [13] or *Dibasterium* Briggs et al. [14] are characterized by having multiple segments within the proximal portion of their chelicerae, and this morphology remains to be integrated within the overall evolutionary scenario of frontal-most appendage evolution.

The other character is the orientation of the appendage, as already outlined by Legg and Vannier (2013). Again, the upward position characterizes isoxiids(*part*)+occacaridids+megacheirans, while amongst dinocaridids and lobopodians the orientation possibly varies from downward to inward directed – but never upward.

Extreme examples of “great appendage” disparity might have been overlooked more recently. The most significant example is probably *Sanctacaris uncata* Briggs and Collins. Originally presented [15] – though with avowed reserve – as having “at least” six pairs of frontal appendages (and therefore related to panchelicerates), a frontal appendage interpretation without formal redescription has been implicitly adopted by some authors of later studies (e.g. Bergström [16] and Budd [17]). The underlying idea would be that the frontal “legs” of *Sanctacaris* represent in fact the multi-branched (peramorphic?) articles of a “great appendage.” The line of evidence for such an interpretation would follow that: among the few specimens available, those frontal elements remain aligned altogether at the anteriormost part of the head; they are associated anteriorly to secondary antennulate appendicles; and, importantly, that other, biramous, cephalic appendages are present in the head, of which the most obvious presents a maxilla-like endopod (S2 Figure). During the review of the present manuscript, Legg [20] published a re-examination of *Sanctacaris* and concluded that the frontal appendages were distinct endopods, and that other elements were various exopods. Without debating the interpretation herein, we chose to examine the significance of a possible assemblage of six limb pairs as rami on a single appendage in the morphospace and thus coded *Sanctacaris* accordingly.

In summary, and in a much broader definition including the frontalmost apparatuses of lobopodians, dinocaridids, “stem bivalved arthropods,” megacheirans and potentially more complex structures based on the same fundamental anatomy, the “great appendage” is a pre-oral appendage bearing developed spines, chiefly on its inner margin. When segmented, the number of podomeres is generally inferior to ca. 15 (*Anomalocaris*, but see e.g. *Paranomalocaris* Wang et al. [21] and *Tamisiocaris* Daley and Peel [3]). In many forms, the spinose differentiation of the appendage is characteristically bipartite. The proximal portion is highly variable in relative size, number of segments and degree of adornment. Proximal segments can bear single or bifid spines of various lengths on their inner margin; single spines can become elongate, slender and somewhat flexible (*Hurdia*, *Schinderhannes*). Spines can bear secondary (or even tertiary, see *Tamisiocaris*) rows of spinnules. Reduced number of elongate proximal spines prefigures – following Haug et al. [2] – the distal finger-bearing portion of forms with stout and segmentally reduced (“short-”) “great appendages,” such as *Yohoia*. On their outer margin, proximal segments can bear additional spines, but those are relatively little developed. Distally, outer spines can be much more developed, as well as being bifid (*Amplectobelua*). The number of developed distal outer spines is three on average, with a fourth, shorter, anteriormost spine sometimes located on the inner margin (*Amplectobelua*, *Laggania* Walcott, *Surusicaris*). Distal inner spines can be poorly developed, or strongly developed when the number of segments is reduced and distal outer spines are absent – in this case, the proximal part ranges from two to no segment. Such developed distal inner spines are from the order of six, seven (*Hurdia*, ?*Sanctacaris*) to two (chelicerates).

1. Chen JY, Waloszek D, Maas A (2004) A new 'great-appendage' arthropod from the Lower Cambrian of China and homology of chelicerate chelicerae and raptorial antero-ventral appendages. Lethaia 37: 3-20.

2. Haug JT, Waloszek D, Maas A, Liu Y, Haug C (2012) Functional morphology, ontogeny and evolution of mantis shrimp-like predators in the Cambrian. Palaeontology 55: 369-399.

3. Vinther J, Stein M, Longrich NR, Harper DAT (2014) A suspension-feeding anomalocarid from the Early Cambrian. Nature 507: 496-499.

4. Dumont HJ, Negrea SV (2002) Introduction to the class Branchiopoda. Dumont HJ (ed.). Leiden: Blackhuys. 388 pp.

5. Scholtz G, Edgecombe GD (2006) The evolution of arthropod heads: reconciling morphological, developmental and palaeontological evidence. Development Genes and Evolution 216: 395-415.

6. Haug JT, Briggs DEG, Haug C (2012) Morphology and function in the Cambrian Burgess Shale megacheiran arthropod *Leanchoilia superlata* and the application of a descriptive matrix. BMC Evolutionary Biology 12: 162.

7. Kuhl G, Briggs DEG, Rust J (2009) A great-appendage arthropod with a radial mouth from the Lower Devonian Hunsrück Slate, Germany. Science 323: 771-773.

8. Hou XG, Bergstrom J, Ahlberg P (1995) *Anomalocaris* and other large animals in the Lower Cambrian Chengjiang fauna of southwest China. GFF 117: 163-183.

9. Dzik J, Lendzion K (1988) The oldest arthropods of the East European Platform. Lethaia 21: 29-38.

10. Daley AC, Budd GE, Caron JB, Edgecombe GD, Collins D (2009) The Burgess Shale anomalocaridid *Hurdia* and its significance for early euarthropod evolution. Science 323: 1597-1600.

11. Sanders HL (1955) The Cephalocarida, a new subclass of Crustacea from Long Island Sound. Proceedings of the National Academy of Sciences of the United States of America 41: 61-66.

12. Sharma PP, Schwager EE, Extavour CG, Giribet G (2012) Evolution of the chelicera: a *dachshund* domain is retained in the deutocerebral appendage of Opiliones (Arthropoda, Chelicerata). Evolution & Development 14: 522-533.

13. Sutton MD, Briggs DEG, Siveter DJ, Orr PJ (2002) The arthropod *Offacolus kingi* (Chelicerata) from the Silurian of Herefordshire, England: Computer based morphological reconstructions and phylogenetic affinities. Proceedings of the Royal Society of London - Biological Sciences 269: 1195-1203.

14. Briggs DEG, Siveter DJ, Sutton MD, Garwood RJ, Legg D (2012) Silurian horseshoe crab illuminates the evolution of arthropod limbs. Proceedings of the National Academy of Sciences of the United States of America 109: 15702-15705.

15. Briggs DEG, Collins D (1988) A Middle Cambrian chelicerate from Mount Stephen, British Columbia. Palaeontology 31: 779-798.

16. Bergström J (1992) The oldest arthropods and the origin of Crustacea. Acta Zoologica 73: 287-291.

17. Budd GE (2002) A palaeontological solution to the arthropod head problem. Nature 417: 271-275.

18. Kuhl G, Rust J (2012) *Captopodus poschmanni* gen. et sp. nov. a new stem-group arthropod from the Lower Devonian Hunsruck Slate (Germany). Arthropod Structure & Development 41: 609-622.

19. Daley AC, Edgecombe GD (2013) Morphology of *Anomalocaris canadensis* from the Burgess Shale. Journal of Paleontology 88:68-91.

20. Legg DA (2014) *Sanctacaris uncata*: the oldest chelicerate (Arthropoda). Naturwissenschaften 101: 1065-1073.

21. Wang Y, Huang D, Hu S (2013) New anomalocaridid frontal appendages from the Guanshan biota, eastern Yunnan. Chinese Science Bulletin 58: 3937-3942.
