# Supplementary material for: Cephalic and Limb Anatomy of a New Isoxyid from the Burgess Shale and the Role of “Stem Bivalved Arthropods” in the Disparity of the Frontalmost Appendage
Source: PLoS One. 2015 Jun 3;10(6):e0124979. doi: 10.1371/journal.pone.0124979 (PMC4454494; doi:10.1371/journal.pone.0124979)
Supplement: S1 Dataset — (DOC) [file pone.0124979.s002.doc]

|  | **1** | **2** | **3** | **4** | **5** | **6** | **7** | **8** | **9** | **10** | **11** | **12** |
| --- | --- | --- | --- | --- | --- | --- | --- | --- | --- | --- | --- | --- |
| *Kerygmachela kierkegaardi* | 0 | 2 | 6 | 3 | 0 | 0 | 0 | 0 | 0 | 0 | 0 | 0 |
| *Aysheaia pedunculata* | 0 | 2 | 4 | 3 | 0 | 0 | 0 | 0 | 0 | 0 | 0 | 0 |
| *Jianshanpodia decora* | 0 | 0 | 5 | 0 | 5 | 0 | 0 | 0 | 0 | 0 | 0 | 0 |
| *Magadictyon haikouensis* | 0 | 0 | 5 | 0 | 5 | 0 | 0 | 0 | 0 | 0 | 0 | 0 |
| *Anomalocaris canadensis* | 15 | 6 | 15 | 0 | 0 | 1 | 0 | 0 | 1 | 0 | 0 | 1 |
| *Amplectobelua stephenensis* | 12 | 3 | 11 | 3 | 0 | 1 | 0 | 0 | 1 | 0 | 0 | 0 |
| *Caryosyntrips serratus* | 12 | 0 | 11 | 0 | 0 | 0 | 0 | 0 | 0 | 1 | 0 | 0 |
| *Peytoia nathorsti* | 11 | 11 | 7 | 3 | 7 | 0 | 1 | 0 | 1 | 0 | 0 | 1 |
| *Hurdia Victoria* | 9 | 8 | 7 | 0 | 7 | 0 | 1 | 0 | 1 | 0 | 0 | 1 |
| *Cassubia infercambriensis* | 7 | 0 | 5 | 0 | 5 | 0 | 0 | 0 | 0 | 0 | 0 | 0 |
| *Opabinia regalis* | 6 | 0 | 6 | 0 | 0 | 0 | 0 | 0 | 0 | 1 | 0 | 0 |
| *Kunmingella maotianshanensis* | 5 | 0 | 4 | 0 | 0 | 1 | 0 | 0 | 0 | 0 | 1 | 0 |
| *Isoxys acutangulus* | 5 | 0 | 4 | 0 | 0 | 0 | 0 | 0 | 0 | 0 | 1 | 0 |
| *Surusicaris elegans* | 5 | 3 | 4 | 3 | 0 | 0 | 0 | 0 | 2 | 0 | 0 | 0 |
| *Occacaris oviformis* | 6 | 0 | 4 | 0 | 5 | 1 | 0 | 0 | 2 | 0 | 1 | 0 |
| *Branchiocaris pretiosa* | 20 | 0 | 0 | 0 | 0 | 0 | 0 | 0 | 0 | 0 | 1 | 0 |
| *Schinderhannes bartelsi* | 9 | ? | 7 | ? | 7 | 0 | 1 | 0 | 1 | 0 | 0 | 1 |
| *Yohoia tenuis* | 6 | 0 | 4 | 0 | 4 | 0 | 0 | 0 | 2 | 0 | 0 | 0 |
| *Fortiforceps foliosa* | 6 | 0 | 4 | 0 | 4 | 0 | 0 | 0 | 2 | 0 | 0 | 0 |
| *Haikoucaris ercaiensis* | 5 | 0 | 3 | 0 | 3 | 0 | 0 | 0 | 2 | 0 | 0 | 0 |
| *Actaeus armatus* | 6 | 0 | 3 | 0 | 3 | 0 | 0 | 1 | 2 | 0 | 0 | 0 |
| *Leanchoilia superlata* | 6 | 0 | 3 | 0 | 3 | 0 | 0 | 1 | 2 | 0 | 0 | 0 |
| *Alalcomenaeus cambricus* | 6 | 0 | 3 | 0 | 3 | 0 | 0 | 1 | 2 | 0 | 0 | 0 |
| *Sanctacaris uncata* | 8 | 0 | 6 | 0 | 6 | 0 | 1 | 0 | 0 | 0 | 0 | 1 |
| *Kiisortoqia soperi* | 15 | 0 | 15 | 0 | 0 | 1 | 0 | 0 | 0 | 0 | ? | 0 |
| *Pycnogonum litorale* | 3 | 0 | 3 | 0 | 2 | 0 | 0 | 1 | 1 | 0 | 0 | 0 |
| *Phalangium opilio* | 3 | 0 | 2 | 0 | 2 | 0 | 0 | 0 | 1 | 0 | 0 | 0 |
| *Tamisiocaris borealis* | 20 | 0 | 20 | 0 | 0 | 1 | 0 | 0 | 1 | 0 | 0 | 1 |
| *Branchia brevis* | 2 | 0 | 2 | 0 | 2 | 0 | 0 | 0 | 1 | 0 | 0 | 0 |
| *Cupiennius foliatus* | 2 | 0 | 1 | 0 | 1 | 0 | 0 | 0 | 1 | 0 | 0 | 0 |
| *Waptia fieldensis* | 10 | 0 | 0 | 0 | 0 | 0 | 0 | 0 | 0 | 0 | 1 | 0 |
| *Lightiella monnotiae* | 6 | 0 | 0 | 0 | 0 | 0 | 0 | 1 | 0 | 0 | 1 | 0 |
| *Nebalia bipes 1* | 4 | 0 | 0 | 0 | 0 | 0 | 0 | 1 | 0 | 0 | 1 | 0 |
| *Nebalia bipes 2* | 18 | 0 | 0 | 0 | 0 | 0 | 0 | 0 | 0 | 0 | 1 | 0 |
| *Lithobius forficatus* | 43 | 0 | 0 | 0 | 0 | 0 | 0 | 0 | 0 | 0 | 1 | 0 |
| *Fuxianhuia protensa* | 15 | 0 | 0 | 0 | 0 | 0 | 0 | 0 | 0 | 0 | 1 | 0 |

For an anatomical overview of "great appendages," on which is based the choice of characters used herein, please see Supporting Information (S1 Comment).

**1. Number of podomeres.**

Interval variable. This character excludes lobopodian taxa.

**2. Number of outer spines.**

Interval variable. Is the count of all identifiable spiny outgrowths of the outer margin of the segments regardless of their degree of development. Applies to lobopodians.

**3. Number of inner spines.**

Interval variable. Is the count of all identifiable spiny outgrowths of the inner margin of the segments regardless of their degree of development. Applies to lobopodians.

**4. Outer 'hand' configuration.**

Interval variable. Is the count of (distal) outer spines conspicuously differentiated into a separate polyfid claw apparatus.

**5. Inner 'hand' configuration.**

Interval variable. Is the count of proximal or distal inner spines conspicuously differentiated and forming a polyfid claw unit.

**6. Paired inner spines.**

Nominal variable (binary). State 1 codes for the presence of paired spines on podomeres

**7. Elongate inner spines.**

Nominal variable (binary). State 1 codes for the presence of remarkably elongate, flexible inner spines, often adorned themselves with secondary teeth (e.g. *Hurdia* condition, see char. 12 below).

**8. Flagellate rami.**

Nominal variable (binary). State 1 codes for the presence of elongate spinose outgrowths of the distal podomeres further prolonged into filamentous minutely segmented flagella (leanchoiliid condition but would apply to many crustaceans as well).

**9. Orientation.**

Nominal variable. 0=frontally or laterally directed, 1=downward directed, 2=upward directed. Although we coded a general condition for *Isoxys acutangulus* in this matrix, a coding for the entire genus would be polymorphic, i.e. (02).

**10. Fused appendage.**

Nominal variable (binary). State 1 applies to the peculiar condition of a joint claw borne by an elongate annulated proboscis in *Opabinia*, which is here also attributed to *Caryosyntrips*.

**11. Rounded terminal segment.**

Nominal variable (binary). The distalmost segment of certain frontalmost appendages lacks a conspicuous outgrowth. State 1 of this character codes for this condition.

**12. Secondary spines.**

Nominal variable (binary). State 1 codes for the presence of secondary processes on the inner spines (typically an anomalocaridid character).
