# Supplementary figures and images for: Cephalic and Limb Anatomy of a New Isoxyid from the Burgess Shale and the Role of “Stem Bivalved Arthropods” in the Disparity of the Frontalmost Appendage
Source: PLoS One. 2015 Jun 3;10(6):e0124979. doi: 10.1371/journal.pone.0124979 (PMC4454494; doi:10.1371/journal.pone.0124979)

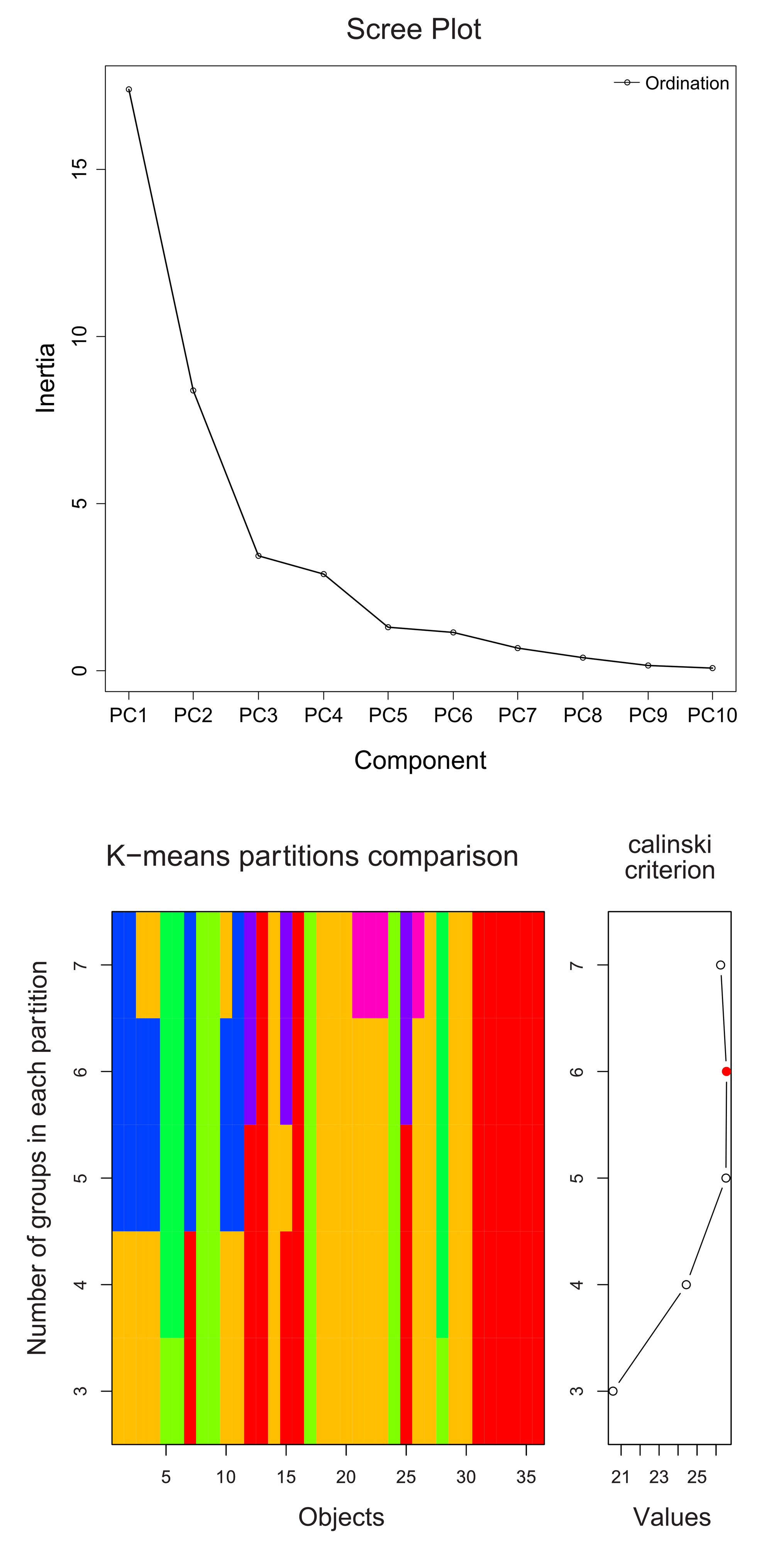

Supplement: S1 Fig — A. Scree plot of the PCoA analysis showing that most of the variance is explained by the first four axes. B. Procedural k-means partitioning on the first four axes of the PCoA set for 3 to 7 groups. The Calinski criterion found an optimum at 6 groups. (TIF) [file pone.0124979.s003.tif]

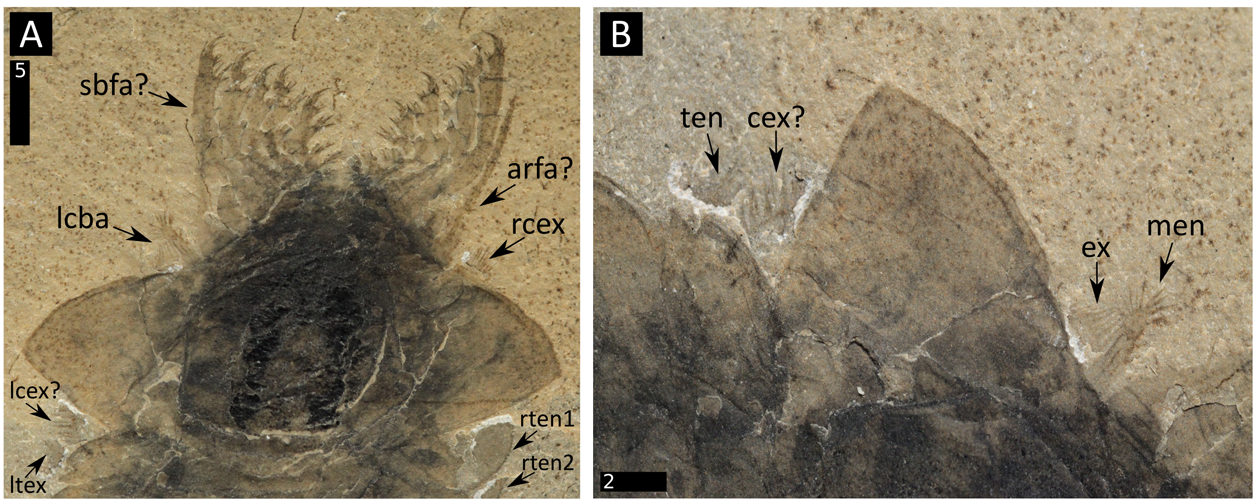

Supplement: S2 Fig — Sanctacaris uncata, part of the holotype (ROM 43502). A. Close-up of the head. Note the presence of at least one differentiated biramous appendage behind the antenniform appendage (see “rcex” and “lcba”) as well as the secondary spinose outgrowths of tridental shape on the frontalmost “legs,” reminiscent of Anomalocaris (e.g. Daley and Edgecombe [97]). Additional preparation reveals thick endopods associated with the trunk segments. B. Close-up of the left pleura in A. The biramous cephalic appendage visible here is composed of an endopod (“men”) whose shape is highly differentiated into a rod bearing distal setae. Close resemblances can be found among the maxillae or maxillules of certain extant crustaceans (e.g. Cephalocarida, see Sanders [123]). Abbr. arfa?: antennular ramus of the frontal appendage?; bsh: broken spinose hand; cex?: cephalic exopod?; ex: exopod; if: internal ‘filament;’ la: left appendage; lcba: left cephalic biramous appendage; lcex?: left cephalic exopod?; ltex: left trunk exopod; mra: margin of right appendage; men: maxilla-like endopod; ra: right appendage; rcex: right cephalic exopod; rtenx: right trunk endopod (1–2); sbfa?: secondary branch of frontal appendage?; sh: spinose hand; ten: trunk endopod. Scale numbers in mm. (TIF) [file pone.0124979.s004.tif]
